# Design, Synthesis, and Application of Immobilized Enzymes on Artificial Porous Materials

**DOI:** 10.1002/advs.202500345

**Published:** 2025-04-30

**Authors:** Lu Ran, Yuan Lu, Li Chen, Mengru He, Zhangshuang Deng

**Affiliations:** ^1^ Hubei Key Laboratory of Natural Products Research and Development College of Biological and Pharmaceutical Sciences China Three Gorges University Yichang 443002 China

**Keywords:** applications, artificial porous materials, enzyme immobilization, multifunctional biological nanoreactors

## Abstract

Enzymes have been recognized as highly efficient biocatalysts, whereas characteristics such as poor stability and single reaction type greatly significantly limit their wide application. Hence, the exploitation of suitable carriers for immobilized enzymes enables the provision of a protective layer for the enzyme, with the capability of chemical and biological cascade catalysis. Among the various immobilization carriers, metal‐organic frameworks (MOFs), covalent organic frameworks (COFs) and hydrogen‐bonded organic frameworks (HOFs) have been emerging as a promising strategy to surpass the inherent instability and other limitations of free enzymes. Specifically, the integration of such artificial porous materials as carriers improves the stability and reusability of enzymes, while simultaneously affording a platform for multifunctional applications. Herein, this review systematically discusses the various preparation strategies and advantages of artificial porous materials, while elucidating the effects of different immobilization methods on enzyme activity. Furthermore, the innovative applications of artificial porous materials as multifunctional carriers in the field of enzyme immobilization fields such as enzyme carriers, photocatalysts, chemical catalysts and sensing are also comprehensively summarized here, thus demonstrating their multifunctional characteristics and promising applications in addressing complex biotransformation challenges.

## Graphical abstract

This review comprehensively summarizes the advances in research for immobilized enzymes on artificial porous materials. It highlights the advantages of bottom‐up strategy, post‐synthesis modification and composite method of metal‐organic frameworks (MOFs), covalent organic frameworks (COFs) and hydrogen‐bonded organic frameworks (HOFs) and emphasizes the impact of different immobilization methods on enzymatic activity. Furthermore, this paper provides a comprehensive overview of the innovative applications of the innovative applications of artificial porous materials as enzyme carriers, photocatalysts, chemical catalysts, and sensing in the field of enzyme immobilization. These findings have important implications for addressing complex biotransformation challenges.

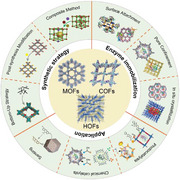

## 1 Introduction

Enzymes are nature's gift to humanity with crucial roles in the evolution of life and the development of society, while also being highly efficient biocatalysts that have been extensively applied in various fields, including asymmetric catalysis, biosensing, tumor therapy, and biosynthesis.^[^ ^1^, ^2^, ^3^, ^4^ ^]^ For instance, lipases can be used for kinetic resolution of secondary alcohols,^[^ ^5^ ^]^ while horseradish peroxidase catalyzes tetramethylbenzidine for colorimetric sensing to detect food toxins.^[^ ^6^ ^]^ Regrettably, the vulnerability of enzymes has severely hampered the breadth of their applications. Significantly, the structural integrity of enzymes is essential for their catalytic function, whereas they are often highly sensitive to environmental circumstances.^[^ ^7^ ^]^ In addition, there are some limitations of enzymes in practical application, such as poor stability, high costs, recovery difficulty, narrow substrate applicability, and a single reaction type.^[^ ^8^, ^9^ ^]^ Particularly, the immobilization technology affords an effective solution to the above problems,^[^ ^10^, ^11^ ^]^ whereby by immobilizing the enzyme on a carrier, the enzyme can be shielded from the interference of the external environment, resulting in the maintenance of the catalytic activity and stability of the enzyme.^[^ ^12^ ^]^ Currently, commonly used carriers (e.g., zeolite molecular sieves, mesoporous silica, and kaolin) have been demonstrated to improve enzyme stability and recovery.^[^ ^13^, ^14^, ^15^, ^16^ ^]^ Nevertheless, the specific surface area of these materials is usually low, which limits the loading capacity of the enzyme and may lead to enzyme leakage during the reaction process,^[^ ^17^, ^18^ ^]^ making the development of suitable enzyme immobilization carriers crucial. Ideal carriers should have structural features that maintain enzyme activity and provide sufficient stability to protect the enzyme, allowing for a high loading capacity of the enzyme.^[^ ^19^ ^]^

**Figure 1:**
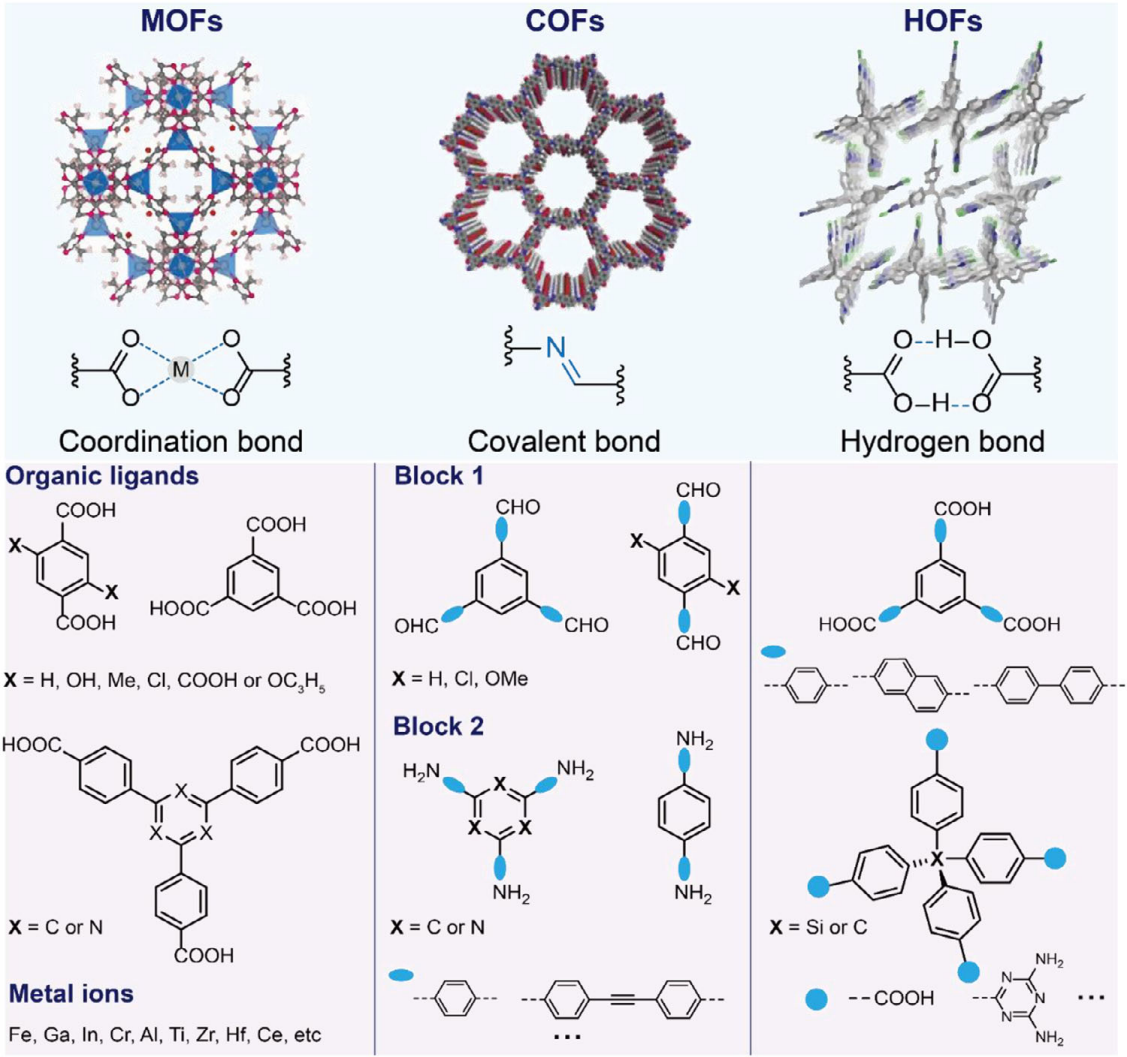
Schematic diagram of structural characteristics of MOFs, COFs and HOFs.

Recently, an emerging class of porous materials has emerged, comprising metal organic frameworks (MOFs), covalent organic frameworks (COFs) and hydrogen‐bonded organic frameworks (HOFs), which feature ordered pore channels, high porosity and high stability.^[^ ^20^ ^]^ In detail, MOFs are composed of organic linkers and metal ions/metal clusters,^[^ ^21^, ^22^, ^23^ ^]^ while COFs are constructed from organic linkers through strong covalent bonds.^[^ ^24^, ^25^ ^]^ In contrast, HOFs are self‐assembled via hydrogen bonding interactions of organic linkers (**Figure** **1**).^[^ ^26^ ^]^ Therefore, these organic porous materials are believed to be ideal carriers for enzyme immobilization for the following reasons: 1) High enzyme‐carrying capacity. High surface area and adjustable porosity of the porous material immobilizes the enzyme on the surface and within the pores of the material, thus ensuring effective enzyme action.^[^ ^27^, ^28^, ^29^ ^]^ 2) High mass transfer efficiency. Enzymes are immobilized in cavities of porous materials, and the framework allows for tight confinement of the enzyme's tertiary structure while still allowing free diffusion of reactants and products. Such efficient mass transfer ensures that the reactants reach the active site of the enzyme quickly, while the products are released rapidly, thereby increasing the efficiency of the catalytic reaction.^[^ ^30^ ^]^ 3) High designability. The high designability of the porous material can be adaptable to the immobilization demands of different enzymes, realizing the diversification of immobilization methods.^[^ ^31^ ^]^ 4) High stability. Porous materials typically have high chemical and mechanical stability, which enhances the chemical and cyclic stability of enzymes and thus extends their functional life.^[^ ^32^, ^33^, ^34^ ^]^ Benefiting from such advantages, porous materials for immobilized enzymes have been successfully applied in a variety of fields.^[^ ^35^ ^]^

**Figure 2:**
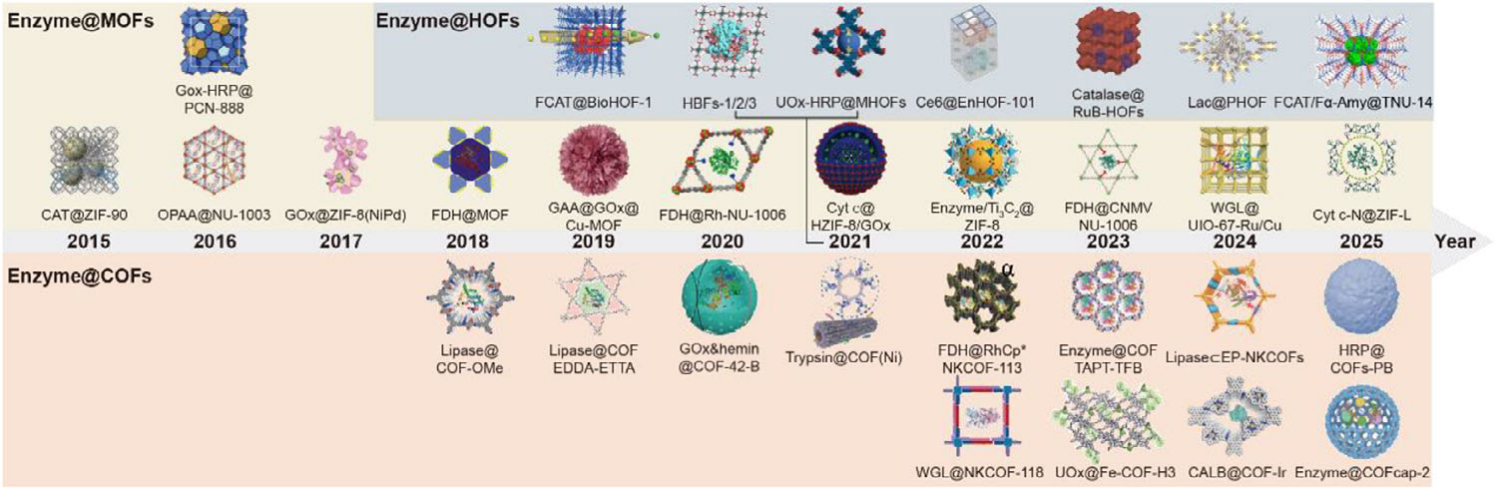
Timeline of artificial porous materials in immobilized enzyme. Image for CAT@ZIF‐90: Reproduced with permission.^[^ ^44^ ^]^ Copyright 2015, American Chemical Society. Image for OPAA@NU‐1003 and GOx‐HRP@PCN‐888: Reproduced with permission.^[^ ^45^, ^46^ ^]^ Copyright 2016, American Chemical Society and Royal Society of Chemistry. Image for GOx‐HRP@ZIF‐8 (NiPd): Reproduced with permission.^[^ ^47^ ^]^ Copyright 2017, Wiley. Image for FDH@MOF and Lipase@COF‐OMe: Reproduced with permission.^[^ ^24^, ^31^ ^]^ Copyright 2018, Science Direct and American Chemical Society. Image for GAA@GOx@Cu‐MOF, Lipase@COF EDDA‐ETTA, and FCAT@BioHOF‐1: Reproduced with permission.^[^ ^48^, ^49^, ^50^ ^]^ Copyright 2019, American Chemical Society and Wiley. Image for FDH@Rh‐NU‐1006 and GOx&hemin@COF‐42‐B: Reproduced with permission. Reproduced with permission.^[^ ^51^, ^52^ ^]^ Copyright 2020, American Chemical Society. Image for Cyt c@HZIF‐8/GOx, Trypsin@COF(Ni), UOx‐HRP@MHOFs, and HBFs‐1/2/3: Reproduced with permission.^[^ ^53^, ^54^, ^55^, ^56^ ^]^ Copyright 2021, Science Direct, American Chemical Society, Wiley and Cell Press. Image for Enzyme/Ti_3_C_2_@ZIF‐8, FDH@RhCp*NKCOF‐113, WGL@NKCOF‐118, Ce6@EnHOF‐101: Reproduced with permission.^[^ ^57^, ^58^, ^59^, ^60^ ^]^ Copyright 2022, American Chemical Society and Wiley. Image for FDH@CNMV‐NU‐1006, Enzyme@COF‐TAPT‐TFB, UOx@Fe‐COF‐H3, and Catalase@RuB‐HOFs: Reproduced with permission.^[^ ^61^, ^62^, ^63^, ^64^ ^]^ Copyright 2023, Royal Society of Chemistry, American Chemical Society, Science Direct, and Wiley. Image for WGL@UIO‐67‐Ru/Cu, Lipase⊂EP‐NKCOF, CALB@COF‐Ir, and Lac@PHOF: Reproduced with permission.^[^ ^65^, ^66^, ^67^, ^68^ ^]^ Copyright 2024, American Chemical Society, Wiley, and American Association for the Advancement of Science. Image for Cyt c‐N@ZIF‐L, HRP@COFs‐PB, Enzyme@COFcap‐2, and FCAT/Fα‐Amy@TNU‐14: Reproduced with permission.^[^ ^69^, ^70^, ^71^, ^72^ ^]^ Copyright 2025, Wiley, Springer Nature, and Royal Society of Chemistry.

Nevertheless, the limitation of reaction types still restricts the extensive application of enzymes. Currently, the combination of chemical catalysis and biocatalysis can address many applications that are difficult to realize with single catalysis, opening a new path for diversified applications of enzymes.^[^ ^36^, ^37^, ^38^, ^39^ ^]^ Another significant advantage of porous materials is their potential for structural functionalization, which provide the possibility for rational design of artificial porous materials. Simultaneously, this characteristic which makes the effective integration of chemical catalysis and biocatalysis possible.^[^ ^40^, ^41^, ^42^, ^43^ ^]^ Exceptionally, artificial porous materials not only enhance enzyme activity but also enable biochemical cascade catalysis, showing great potential as multifunctional carriers. Unfortunately, current strategies for enzyme immobilization using these versatile materials have been relatively restricted. In order to fully utilize the potential of porous materials for enzyme immobilization applications, there is an urgent need to design rational materials to overcome the existing challenges. Therefore, it is of significant importance to construct multifunctional bio‐nanoreactors for applications in various fields. Up to date, the related works about the immobilization of enzymes by artificial porous materials have been reported continuously (**Figure** **2**). However, there are still rare about systematic review on the immobilization of enzymes on artificial porous materials and their applications in various fields. Therefore, a comprehensive review would be needed to systematically summarize the changes in enzyme performance and the expansion of applications after immobilizing enzymes on artificial porous materials.

This review comprehensively outlines the latest research advances in the use of artificial porous materials as multifunctional carriers for immobilizing enzymes. Furthermore, various preparation strategies of artificial porous materials and the changes in material properties after modification are discussed in detail. Additionally, the specific effects of different immobilization methods on enzyme activity and the key interactions between the enzyme and the carrier are also explored. Besides, the application of MOF‐, COF‐ and HOF‐based artificial porous materials as multifunctional enzyme immobilization carriers, including their roles as enzyme carriers, photocatalysts, chemocatalysts, and sensing, is extensively reviewed. In the end, the challenges encountered in the design and application of artificial porous materials as enzyme immobilization carriers are summarized.

## 2 Strategies for Constructing Artificial Porous Materials

**Figure 3:**
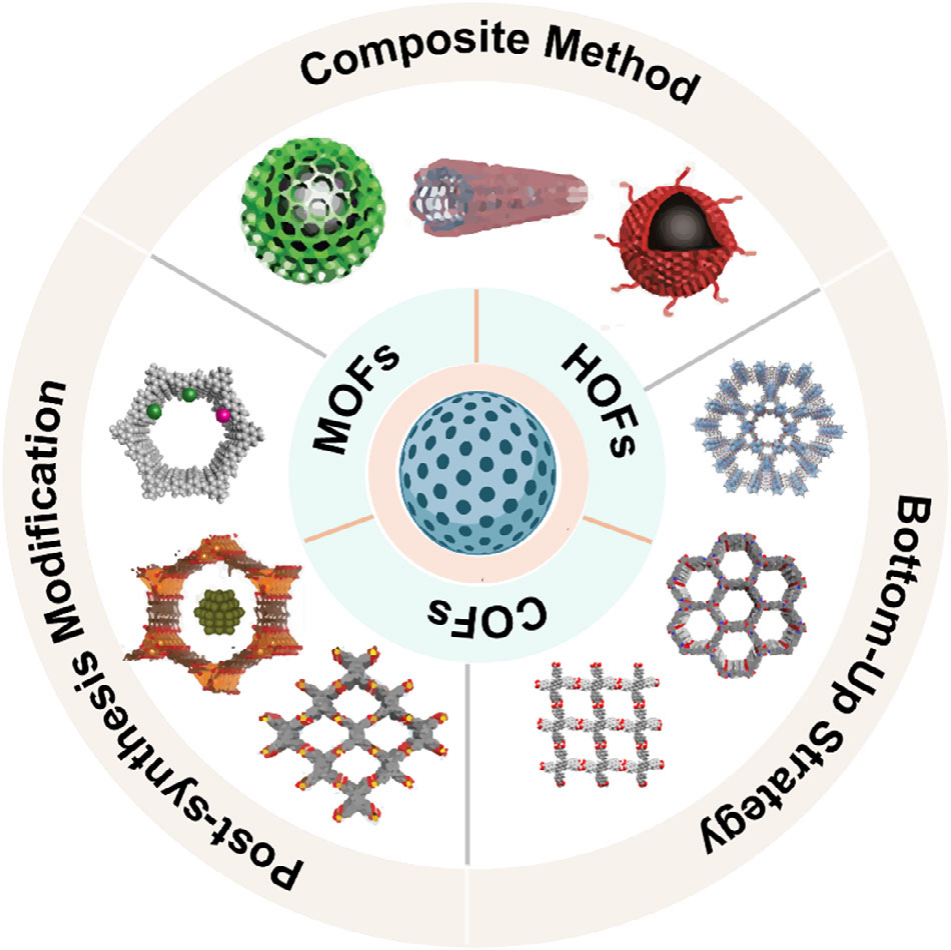
Construction strategy of artificial porous materials.

The structural diversity and tunable functionality of porous materials enable them to facilitate complex catalytic processes. Researchers have employed a variety of strategies and methods to design multifunctional porous materials.^[^ ^73^, ^74^, ^75^ ^]^ Specifically, the construction of artificial porous materials can be categorized as follows: bottom‐up strategy, post‐synthetic modification, and composite approach (**Figure** **3**).

### 2.1 Bottom‐Up Strategy

Basically, the bottom‐up strategy is considered to be the simplest and most commonly used technique for constructing artificial porous materials, with the advantages of chemical and thermal stability as well as homogeneous distribution of active sites in the artificial porous materials. The versatility of artificial porous materials stems mainly from the choice of building blocks, whose functional adjustability includes: 1) Selection of specific organic ligands (porphyrin, pyrene, etc.) to give the material optoelectronic properties;^[^ ^76^, ^77^, ^78^ ^]^ 2) Fine‐tuning of the building blocks by halogenation, cyanation, hydroxylation, or changing the substitution position to achieve precise control over the functionality of the artificial porous material;^[^ ^79^ ^]^ 3) The connection pattern and type of bonding of the components give the material a high degree of stability.^[^ ^80^ ^]^ For example, Jin and co‐authors successfully developed a novel mixed‐ligand MOF by integrating 5,15‐di (*p*‐benzoato)porphyrin (H_2_DPBP) and naphthalene diimide into the MOF structure via a one‐pot synthesis strategy. With this novel approach, the MOF is assigned the dual functions of light‐harvesting and photocatalysis. Specifically, the material was used for amine coupling reactions under aerobic conditions and revealed efficient photocatalytic performance for various amine‐based substrates.^[^ ^81^ ^]^ Furthermore, Chen et al. synthesized a series of classical MOF photocatalysts that exhibited superior stability. Photocatalytic experiments have shown that porphyrin‐containing MOFs exhibited excellent photocatalytic properties for various halogen atom transfer reactions, including dehalogenation, hydroalkylation, and polyfluoroarylation.^[^ ^74^ ^]^

**Figure 4:**
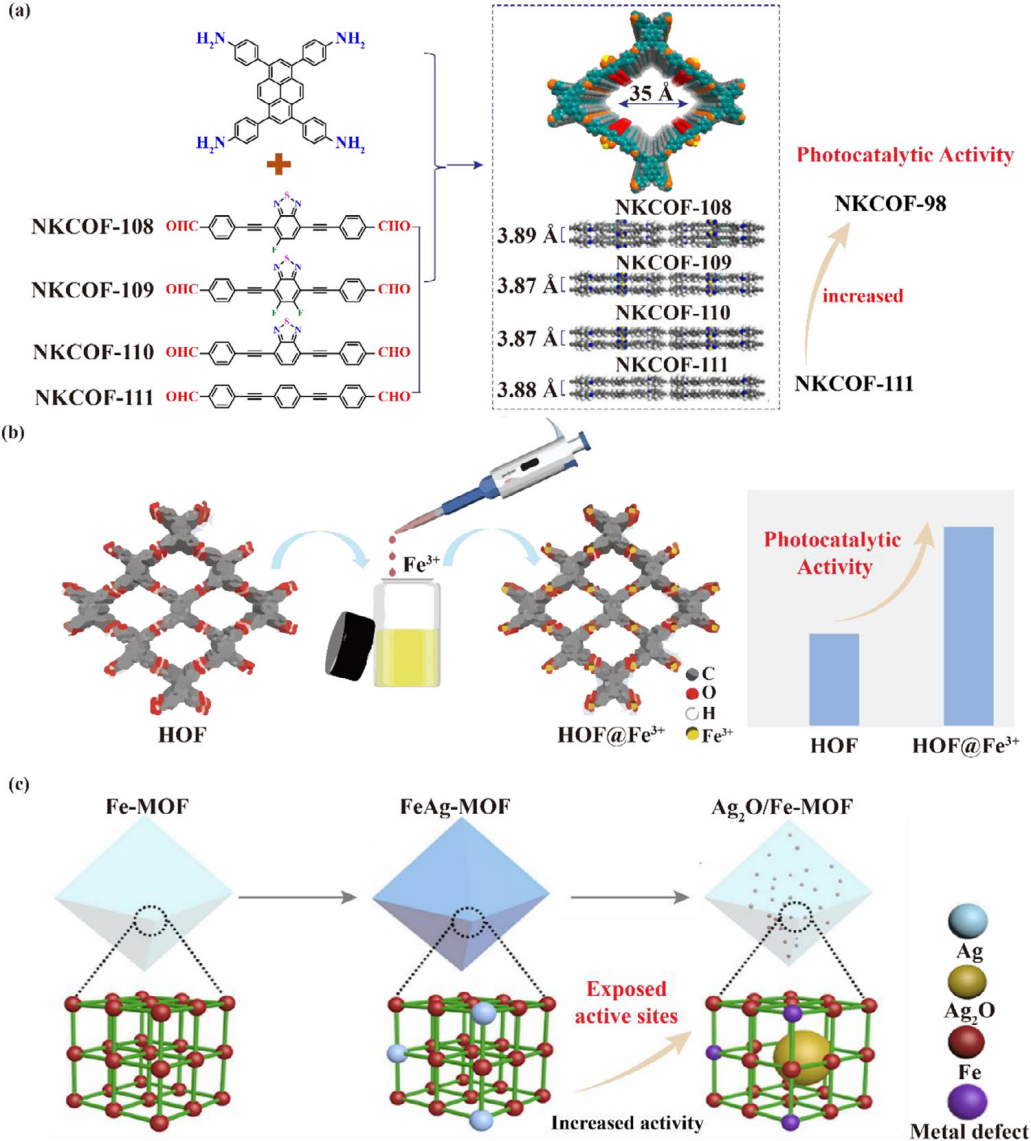
a) Chemical structure of NKCOFs with photocatalytic performance. Reprinted with permission.^[^ ^82^ ^]^ Copyright 2021, American Chemical Society. b) Schematic diagram of coordination Fe^3+^ onto HOFs. Reproduced with permission.^[^ ^92^ ^]^ Copyright 2023, Wiley. c) Schematic diagram for the preparation of Ag_2_O/Fe MOF composite material. Reproduced with permission.^[^ ^97^ ^]^ Copyright 2022, Elsevier.

Both COFs and HOFs can also achieve their multifunctionality by tuning the organic ligands. Chen's group designed a series of COFs with different photocatalytic properties by fine‐tuning the COF monomers (**Figure** **4**a),^[^ ^82^, ^83^ ^]^ which were subsequently applied to the photocatalytic hydrogen evolution reaction. These mesoporous COF materials exhibited significant differences in the charge density distribution of the lowest unoccupied molecular orbital (LUMO) and the highest occupied molecular orbital (HOMO) within their unit cells. Remarkably, the LUMO and HOMO orbitals of NKCOF‐113‐M displayed a more homogeneous electron distribution property compared to the other two COF materials as the distance between the acceptors varied. As a result, the nonlocalized π‐electrons in NKCOF‐113‐M possessed higher mobility in the π‐conjugation plane, which promoted the separation and transport of photogenerated charge carriers.^[^ ^82^ ^]^ Consequently, this effort laid the foundation for the development of an integrated COF platform with dual functions of photocatalysis and enzyme carrier. Besides, Farha's research group synthesized a series of HOF materials by choosing different organic monomers, called HOF‐100, HOF‐101 and HOF‐102. Among them, HOF‐102 possessed an excellent photochemical detoxification ability for mustard gas simulant.^[^ ^75^ ^]^ With this strategy, both the high crystallinity of the constructed artificial porous materials and the uniform distribution of the active sites were ensured, which improved the utilization efficiency of the materials.

### 2.2 Post‐Synthesis Modification

Another strategy to prepare artificial porous materials is through post‐synthetic modification.^[^ ^84^, ^85^ ^]^ Upon modification, various functional groups can be incorporated into the framework of the porous materials, such as coordinating certain metals or linking organic small molecules with catalytic sites, thus strengthening the intrinsic properties of the porous material.^[^ ^86^ ^]^ Post‐synthesis functionalization of porous materials can express outstanding activity in different catalytic reactions, suggesting that this strategy is an effective and straightforward approach to functionalization. In 2018, Lin et al. successfully anchored Ir(III) and Ni(II) in a mesoporous MOF to create a composite system in which Ir(III) acted as a photoredox and Ni(II) as an organic metal catalyst. Under light irradiation, this co‐catalytic system significantly promoted the cross‐coupling reaction between aryl iodides and thiols. Control experiments revealed that the original MOF was unable to catalyze the reaction, highlighting the critical roles of Ir(III) and Ni(II) in the catalytic process. Simultaneously, this prepared material demonstrated excellent cyclic stability and could be reused for six cycles without loss of catalytic activity.^[^ ^87^ ^]^

Presently, metal coordination is a widely adopted method for post‐synthesis modification of COFs and HOFs, which allows metal ions to form coordination bonds with organic ligands in COFs, thus enabling control over the structure and function of the materials.^[^ ^88^, ^89^, ^90^ ^]^ This post‐synthesis strategy not only improves the stability and catalytic properties, but also introduces specific metal centers to catalyze specific chemical reactions. For instance, Ballesté et al. coordinated [(dF(CF_3_)ppy)_2_Ir‐μ‐Cl]_2_ and NiCl_2_·glyme to a COF containing triamine 1,3,5‐tris (4‐aminophenyl) benzene (TAPB) and 4,4′‐(1,10‐phenanthroline‐3,8‐diyl)dibenzaldehyde, resulting in a photo‐mediated Csp^3^‐Csp^2^ cross‐coupling reaction.^[^ ^91^ ^]^ In addition, Lu et al. constructed a porous material, HOFs@Fe^3+^, to immobilize Fe^3+^ on HOFs through electrostatic and coordination interactions (Figure 4b). Subsequently, it was applied to the adsorption and degradation of bisphenol A (BPA) with a high adsorption rate of 452 mg g^−1^. In particular, HOFs@Fe^3+^ could complete the adsorption and photodegradation of 50 ppm BPA within 20 min. The introduction of Fe^3+^ effectively solved the problem of poor photogenerated carrier mobility of HOFs, which improved the photocatalytic activity of the material.^[^ ^92^ ^]^ Resultantly, well‐designed porous materials not only contribute to metal coordination, but also offer broader prospects for subsequent catalytic applications. Although post‐synthesis modification strategies can introduce excellent active sites to porous materials, but they may also diminish their crystallinity, in addition to the challenge of ensuring that the post‐modified groups are uniformly distributed on the material framework.

### 2.3 Composite Method

Combining nanomaterials with porous materials using covalent bonding, hydrogen bonding and π–π interactions can perfectly integrate the physicochemical properties, synergistic effects and multiple functionalities of different materials, which can improve the performance of artificial porous materials and expand their applications.^[^ ^93^ ^]^ Based on these advantages, porous materials can be combined with nanomaterials (graphene, carbon nitride, and metal oxides, etc.) to address their inherent limitations, thereby improving the electrical conductivity, light‐absorption capacity, and catalytic activity of artificial porous materials.^[^ ^94^, ^95^, ^96^ ^]^ In specific applications, each material has its inherent advantages and disadvantages, while composite methods can integrate the properties of different materials to achieve complementary advantages, resulting in new composite porous crystalline materials with better performance than single‐component materials. In 2022, Qin et al. prepared Ag_2_O/Fe‐MOF composites by in situ photochemical synthesis, which were artificial porous materials that could be used for the photocatalytic conversion of microplastics (MPs) and the generation of H_2_. The developed photochemical design strategy was able to generate uniformly distributed Ag_2_O particles in the pores of MOFs, thus exposing a large number of active sites, with improved light‐absorption range and charge‐transfer rate in Ag_2_O/Fe‐MOF composites (Figure 4c). As a result, the Ag_2_O/Fe‐MOF photocatalysts exhibited high photocatalytic performance in converting MPs and generating H_2_. Besides, it was also found that the Ag_2_O/Fe‐MOF photocatalysts could highly selectively convert MPs into high value‐added products.^[^ ^97^ ^]^ Similarly, composites of COFs and HOFs have been shown to improve material properties.^[^ ^98^, ^99^ ^]^ Nonetheless, the combination of multiple materials is still in its infancy and faces several unresolved challenges, such as difficulties in controlling crystallinity and porosity, inability to predict the properties of composites, the presence of structural defects and so on.

Three primary methods for the preparation of artificial porous materials have been comprehensively described and can be comparatively analyzed: i) The bottom‐up strategy has been widely applied in the fabrication of artificial porous materials due to its advantages of high stability, simple operation, and controllable structure. ii) Post‐synthetic modification allows for more precise control over the structural properties of the materials than bottom‐up strategies, resulting in the production of artificial porous materials with multiple catalytically active sites. iii) In contrast to material composite methods, the post‐synthetic modification strategy facilitates substrate mass transfer, offering a more promising and efficient approach to chemical catalysis. Overall, the application of these artificial porous materials for enzyme immobilization holds the potential to improve enzyme stability and diversify applications.

## 3 Artificial Porous Materials as Enzyme Carriers

### 3.1 Performance of Enzyme Immobilization

After the immobilization of enzymes on artificial porous materials, the conformation, activity, and catalytic performance of the enzymes are significantly influenced by the characteristics of the carriers, as detailed below: 1) HOF minimizes interference with the natural conformation of enzymes due to its weak hydrogen‐bonding interactions than that of MOF and COF immobilized enzymes, thereby better preserving the accessibility to the active sites of the enzyme and enhancing catalytic efficiency. Additionally, the flexible pore structure of HOF can adapt to the size variations of enzymes, avoiding the conformational restrictions imposed by rigid carriers. 2) COF exhibits superior water stability and chemical stability, enabling the preservation of the natural activity of enzymes in aqueous environments compared with HOF and MOF immobilized enzymes. Furthermore, the highly ordered structure and stable covalent bonding characteristics of COF contribute to excellent performance retention during multiple cycles of use, significantly improving the recyclability of immobilized enzymes. 3) Compared to COF and HOF immobilized enzymes, MOF provides efficient mass transfer channels for substrates and products due to its hierarchical pore structure (e.g., coexistence of micropores and mesopores) and tunable pore sizes, thereby significantly enhancing the efficiency of catalytic reactions. Moreover, the high surface area and rich surface chemistry of MOF enable higher enzyme loading capacities, further enhancing its potential in catalytic applications. MOFs, COFs and HOFs each offer unique advantages in enzyme immobilization: Selecting the appropriate carrier based on specific application requirements can maximize the performance and application potential of immobilized enzymes.

### 3.2 Strategies for Integrating Enzymes

**Figure 5:**
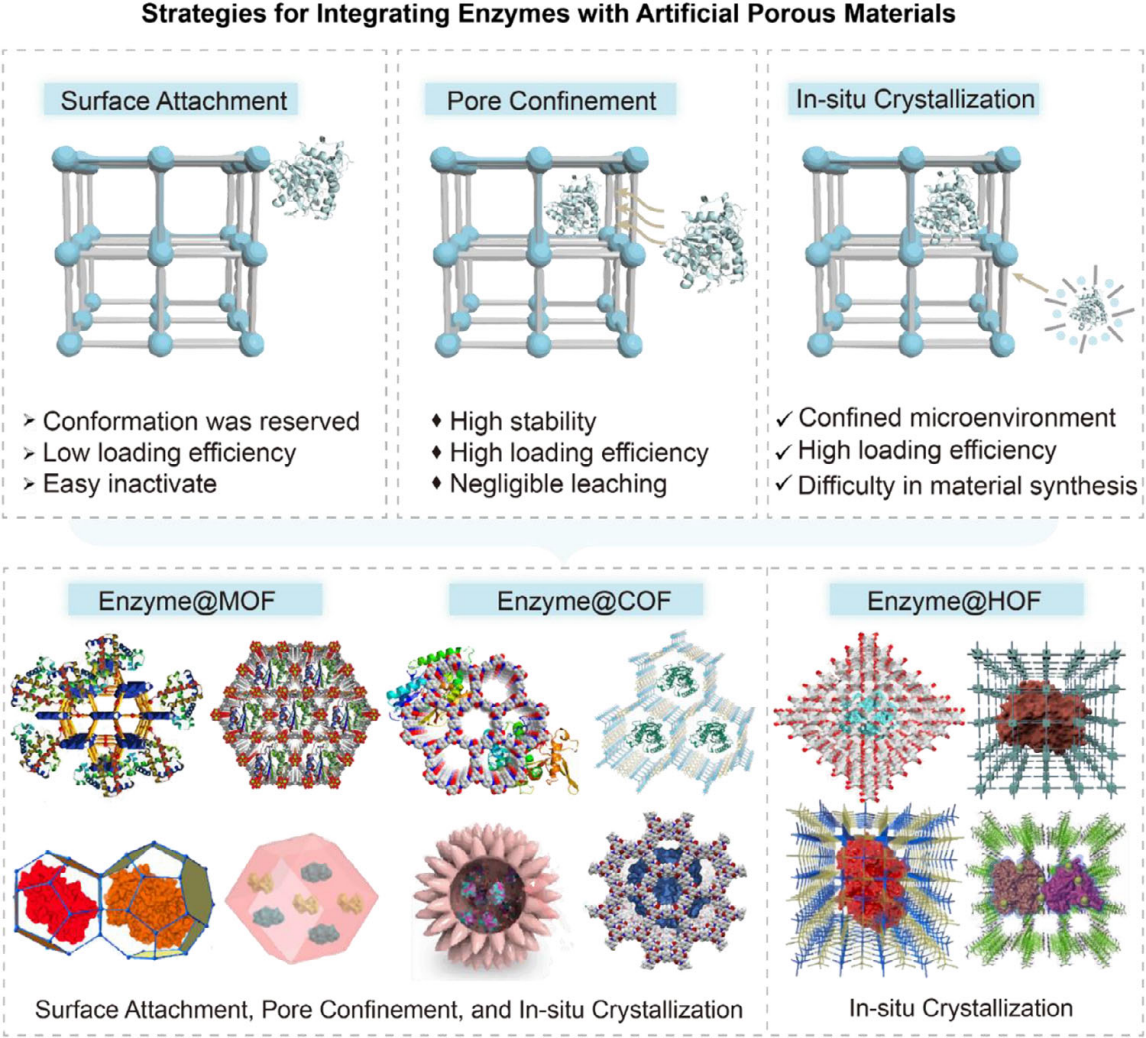
Strategies for enzyme immobilization using porous materials.

As emerging artificial porous materials, MOFs, COFs and HOFs have been widely exploited for enzyme immobilization.^[^ ^31^, ^100^, ^101^, ^102^ ^]^ Among them, MOFs, COFs and HOFs provide diverse enzyme immobilization strategies, including surface attachment, pore confinement, and in situ crystallization. By tuning the pore size, surface chemistry and structural design of the materials, above approaches achieve efficient enzyme loading and stable immobilization. Each approach has unique advantages and limitations (**Figure** **5**).

#### 3.2.1 Surface Attachment

Surface adsorption via non‐covalent interactions is a common and straightforward strategy for anchoring enzymes to pre‐synthesized artificial porous frameworks, typically requiring neither complex experimental procedures nor harsh conditions, while preserving the natural conformation of enzymes. This approach relies mainly on intermolecular forces, including hydrophilic and hydrophobic interactions, van der Waals forces, electrostatic forces, and π–π interactions.^[^ ^103^, ^104^, ^105^, ^106^, ^107^ ^]^ To improve the efficiency of enzyme immobilization, functionalization of porous material surfaces is an effective strategy. For example, the hydrophobic UiO‐66 was prepared by polydimethylsiloxane coating and adsorbed *Aspergillus niger* lipase (ANL) through hydrophobic interactions. Compared with ANL/UiO‐66, ANL/UiO‐66‐PDMS exhibited higher enzyme activity and activity recovery.^[^ ^32^ ^]^ The charged organic dye fluorescein isothiocyanate can be anchored to the enzyme to form effective strong π–π interactions and hydrogen bonds with the organic linkers of MOFs.^[^ ^108^ ^]^ Covalent organic framework (Cu/Fe‐COFs) based on Cu^2^⁺/Fe^3^⁺ were constructed by the introduction of metals (e.g., Cu^2+^/Fe^3+^). Through electrostatic interactions between the surface charge of metal ions and GOx, efficient surface adsorption of glucose oxidase (GOx) was realized.^[^ ^109^ ^]^

Surface immobilization through noncovalent interactions, although easily achieved, may suffer from poor binding stability, susceptibility to environmental interference and limited reusability due to the reliance on weak intermolecular forces, whereas covalent linkage between the functional groups of artificial porous materials and enzymes can effectively address these shortcomings. Various functional groups have been introduced on the surface of artificial porous materials as needed. Common functional groups include hydroxyl, carboxyl, epoxy and azide groups, which can interact specifically with the active groups in the enzyme molecule (e.g., thiol‐amino and carboxyl groups) to achieve targeted binding between the enzyme and the material.^[^ ^110^, ^111^, ^112^ ^]^ To further improve the immobilization efficiency, some functional additives such as 1‐ethyl‐3‐(3‐dimethylaminopropyl)carbodiimide (EDC), *N*‐hydroxysuccinimide (NHS), and dicyclohexyl carbodiimide (DCC), can be introduced into the artificial porous materials, which are capable of activating or functionalizing the amino or carboxyl groups on the carrier and thus enhancing their reactivity with amino groups of the enzyme. Covalent immobilization of organophosphorus hydrolase (OPH) was achieved by NHS/EDC activation of the carboxyl group on COF (TTB‐DHBD/TTB‐DHBD) through the condensation reaction between the carboxyl group and the amino group.^[^ ^113^ ^]^ The amino functional group exposed on the surface of MIL‐101‐NH_2_ can be chemically modified using N‐succinimidyl‐3‐(2‐pyridyldithio)propionate, which can be used to graft the disulfide bond to the surface of MOF, thus realizing covalent immobilization of ovalbumin through disulfide thiol exchange.^[^ ^114^ ^]^

As known, this method is simple and facile to operate on different MOFs or COFs carriers. Nevertheless, the surface‐immobilized enzyme remains directly exposed to the outside world, leading to enzyme inactivation. Moreover, the surface immobilization ignores the inherent high porosity of materials, which results in low loading efficiency.

#### 3.2.2 Pore Confinement

By incorporating enzyme into the size‐matched channels of artificial porous materials, high loading capacity can be realized and effective protection for enzymes is offered under harsh conditions.^[^ ^115^, ^116^ ^]^ In addition, artificial porous materials have great design flexibility in terms of topology, pore size and surface chemistry, which can achieve tight confinement and stabilization of the fragile structures of enzymes and effectively reduce problems such as inactivation and leakage.^[^ ^117^ ^]^ What's more, the ordered channel structure of the artificial porous material creates a unique internal space for the enzyme, which facilitates complete encapsulation of the enzyme and develops a favorable microenvironment in the pores, thus further enhancing the activity and stability of the enzyme.^[^ ^118^ ^]^

In the pore confinement strategy, the pore size and pore environment of the artificial porous materials constitute the key factors affecting the immobilization efficiency, in which the interactions generally include physical adsorption or covalent linkage,^[^ ^66^, ^119^ ^]^ with this strategy being widely used for enzyme immobilization in artificial porous materials. Taking advantage of the superior porosity and customizable pore structure of MOFs, the researchers investigated the size‐selective effect of mesoporous MOFs on enzyme substrates, the infiltration behavior of immobilized enzymes, and the host‐guest interaction between MOFs and enzymes.^[^ ^119^ ^]^ For example, Farha's group constructed MOFs (NU‐1003, PCN‐600, and CYCU‐3) for pore encapsulation of cutinase, revealing that MOFs with different pore structures are capable of diverse enzyme adsorption.^[^ ^120^ ^]^ In order to improve the biocatalytic efficiency, the pore size distribution of COFs was systematically analyzed using density functional theory (DFT) calculations before immobilizing the enzyme by Ma and co‐authors. The results demonstrated that: 1) The narrow triangular channel (13.9 Å) in the COF effectively excludes biomolecules while allowing free diffusion of reactants and intermediates, thus preventing enzymes from clogging or partially clogging the pores and significantly reducing the mass transfer efficiency; 2) The relatively large mesoporous channels (38.5 Å) offer an optimal spatial environment for the ideal distribution of the enzyme, ensuring efficient loading and stable immobilization. This study presented a theoretical basis for optimizing the distribution of enzyme within the COFs and enhancing its catalytic effect.^[^ ^49^ ^]^ Furthermore, studies have revealed that the covalent immobilization of enzyme in COF pores results in minimal leaching and excellent stability.^[^ ^118^ ^]^ In addition, the 3D mesoporous COFs is suitable as a carrier for enzyme immobilization due to its efficient molecular diffusion and accessibility to the enzyme.^[^ ^121^ ^]^

#### 3.2.3 In Situ Crystallization

An alternative approach for enzyme immobilization is to utilize the mild synthetic conditions of artificial porous materials to create a stable scaffolding framework around the enzyme by in situ crystallization, resulting in versatile enzyme‐based biocomposites. Such approach can encapsulate enzymes with larger pore sizes than the material, thus minimizing enzyme leakage and addressing the limitations of surface immobilization and pore penetration, while providing protection for the enzyme under harsh conditions.^[^ ^18^, ^122^ ^]^ As the most classical material among MOFs, ZIF‐8 can be assembled in situ with enzymes in aqueous solution at room temperature.^[^ ^123^, ^124^, ^125^, ^126^ ^]^ For example, Song et al. successfully demonstrated that co‐precipitation of cytochrome c (Cyt c) in ZIF‐8/TiO_2_ overcame the limitations of the microporous structure for the immobilization of larger‐sized enzymes. In Cyt c, the Met 80 residue is coordinated to the sixth axial position of the heme iron, forming a stable hexa‐coordinated Fe‐S bond in the geometry. However, the Fe‐S bond between Met 80 and iron is susceptible to breakage due to immobilization by the material, which leads to stronger coupling between the nearby aromatic amino acids and the π–π* transition of heme, thus enhancing the catalytic activity of the enzyme.^[^ ^127^ ^]^ Furthermore, immobilization of the dual enzymes (ADH and GDH) in ZIF‐8 via pore encapsulation catalyzed the conversion of ketones to alcohols, resulting in chiral alcohols with high yields (up to 99%) and superior enantioselectivity (>99%). And this study applied them to the synthesis of marketed drugs. In particular, this material has been applied to the synthesis of commercially available drugs.^[^ ^128^ ^]^

Recently, a new type of biocomposites formed by self‐assembly of COF monomers and enzymes has attracted extensive attention from many researchers.^[^ ^62^, ^129^, ^130^ ^]^ Generally, the preparation of COFs requires harsh conditions such as high temperature, organic solvents and acid catalysts, whereas by improving these conditions, COFs can be tailored as enzyme inclusion complexes, represented mainly by Chen. The resulting biocatalysts have excellent reusability and stability with large‐scale preparation, a strategy that is applicable to a wide range of COFs and enzymes.^[^ ^131^ ^]^ Importantly, immobilized enzymes greatly improved their activity and stability compared to free enzymes under harsh conditions.

Different from traditional MOFs and COFs, the synthesis of HOFs is typically carried out in aqueous solutions or under mild conditions, avoiding harsh environments such as high temperatures, strong acids or bases, which better protects the native conformation and biological activity of the enzyme.^[^ ^50^, ^132^ ^]^ Amino acid residues on the enzyme surface can form hydrogen bonds with hydrogen bond donors or acceptors in HOFs, thus realizing the immobilization of enzyme.^[^ ^133^ ^]^ For instance, Gangfeng Ouyang et al. used 1,3,6,8‐tetrakis (*p*‐benzoic acid) pyrene (H4TBAPy), 6,6′,6′′,6′′′‐(Pyrene‐1,3,6,8‐tetrayl)tetrakis(2‐naphthoic acid) (H4PTTNA) and 3,3′,5,5′‐Tetrakis‐(4‐carboxyphenyl)‐1,10 ‐biphenyl (H4TCBP) as building blocks for protein‐directed assembly by strong π–π stacking, denoted as HBF‐1, HBF‐2 and HBF‐3, respectively. Furthermore, DFT calculations were performed to determine the free energy changes associated with the formation of hydrogen bonds between H4TBAPy and the amide N–H residue (surface residue, ΔG = −357.7 kcal mol^−1^), H4TBAPy and the COOH residue (surface residue, ΔG = −357.8 kcal mol^−1^), as well as H4TBAPy and the N‐H group (peptide backbone, ΔG = 5.3 kcal mol^−1^). As a result, the COOH and N‐H residues of proteins provide strong hydrogen bonding sites that greatly facilitate the formation of hydrogen‐bonded organic frameworks (HOFs). Compared to free enzymes, biocomposites offer significant advantages in biocatalysis.^[^ ^56^ ^]^ In addition, HOFs can also be combined with other materials for enzyme immobilization. Sun et al. employed a polyelectrolyte‐assisted encapsulation approach to co‐immobilize four oxidoreductases and NAD(P)H cofactors in a cascade reaction, thus replacing the direct interaction between enzymes and HOF monomers to prevent enzyme destruction by HOF monomers.^[^ ^134^ ^]^

#### 3.2.4 Other Approaches

**Figure 6:**
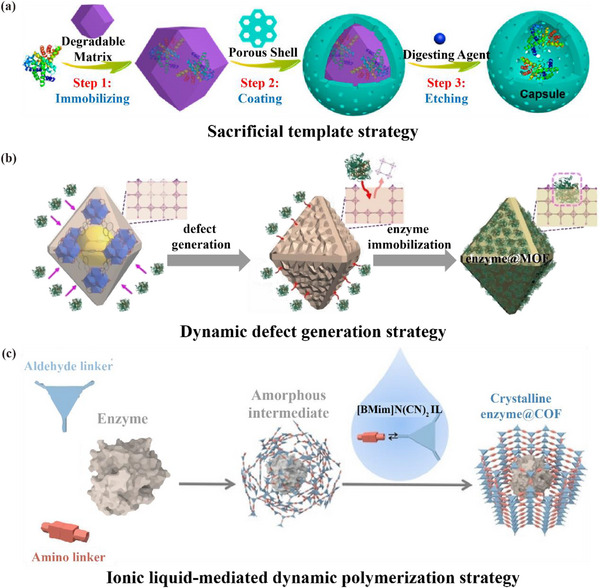
a) COF encapsulating enzyme was prepared by sacrificial template method. Reproduced with permission.^[^ ^52^ ^]^ Copyright 2020, American Chemical Society. b) MOF encapsulating enzyme was prepared by dynamic defect generation strategy. Reproduced with permission.^[^ ^138^ ^]^ Copyright 2023, Wiley. c) The immobilization of Enzymes in COF by ionic liquid‐mediated dynamic polymerization. Reproduced with permission.^[^ ^139^ ^]^ Copyright 2024, Wiley.

Artificial porous materials can also be immobilized with enzymes via sacrificial templates, dynamic defect generation strategies, and ionic liquid‐mediated dynamic polymerization strategies. The sacrificial template strategy involves nucleation and growth of biocomposites as the core to form hollow microcapsules, which is a template‐assisted method that provides a straightforward and scalable approach to the synthesis of hollow microcapsules.^[^ ^135^ ^]^ Under the limited synthesis conditions of COFs, the use of MOFs as a sacrificial template avoids enzyme inactivation during assembly and prevents COF structure collapse. Chen et al. developed hollow COF microcapsules for encapsulating enzyme, providing a wide internal environment to maintain the conformational freedom of the enzymes (**Figure** **6**a).^[^ ^52^ ^]^ Notably, the template‐directed artificial porous materials strategy has been applied in the fields of biosensing, biocatalysis, and biotherapy.^[^ ^52^, ^136^, ^137^ ^]^ Furthermore, the dynamic defect strategy utilizes enzymes containing amino acid residues as “ligands” that competitively coordinate with the metals in the MOFs to create defects, thus enabling enzyme immobilization during the dissociation equilibrium of the MOFs (Figure 6b).^[^ ^138^ ^]^ With this approach, the limitations of conventional immobilized MOFs under harsh synthesis conditions are overcome. Additionally, a small amount of ionic liquid can be introduced into the COF‐based enzyme immobilization process to promote the formation of highly crystalline biocomposites through an ionic liquid‐mediated dynamic polymerization strategy (Figure 6c).^[^ ^139^ ^]^

Different immobilization strategies have their respective advantages and limitations. Therefore, the selection of an appropriate immobilization strategy requires comprehensive consideration of enzyme properties, reaction conditions and application requirements to maximize the catalytic properties and application potential of enzymes.

## 4 Application of Immobilized Enzymes on Artificial Porous Materials

The immobilization of enzymes on functionally specific artificial porous materials leads to surprising performance improvements, thus expanding the range of applications and solving many applications that are difficult to achieve with a single catalyst. In addition, it offers many advantages over homogeneous catalysts, such as low cost, high stability and recyclability. This section will summarize the role of functional artificial porous materials in enzyme immobilization and the corresponding applications (**Table** **1**).

**Table 1:** Summary of the immobilized enzyme on artificial porous materials and its applications.

Artificial porous materials	Preparation strategy	Enzyme	Immobilization method	Application	Refs.
CNMV‐NU‐1006	post‐synthesis modification	FDH	pore confinement	biocatalysis	[61]
UiO‐67‐Ru/Cu	post‐synthesis modification	WGL	pore confinement	biocatalysis	[65]
Fe‐ZIF‐8	bottom‐up Strategy	GOx	in situ crystallization	biocatalysis	[182]
Ce/Zr‐UiO‐66	composite method	HRP	surface attachment	biosensor	[166]
MOF@Ag	composite method	GOx	in situ crystallization	therapy	[157]
ZIF‐8/Alg	bottom‐up Strategy	Tyr/ Lac/ GOx	in situ crystallization	biosensor	[183]
Zr‐MOF	bottom‐up Strategy	FDH/ Ferredoxin‐NADP^+^ reductase	pore confinement	biocatalysis	[153]
HZIF‐8	post‐synthesis modification	HRP/ Lipase	in situ crystallization	biocatalysis	[184]
NU‐1510‐Cr	bottom‐up Strategy	ADH/ FDH	pore confinement	biocatalysis	[185]
NKCOF‐118(Zn)	bottom‐up Strategy	WGL	pore confinement	biocatalysis	[59]
Ru/Rh‐COF	post‐synthesis modification	ADH	pore confinement	biocatalysis	[156]
Ni‐TpBpy COF	post‐synthesis modification	β‐Glu	pore confinement	biocatalysis	[186]
porphyrin‐based COF	bottom‐up Strategy	GOx/CAT	surface attachment	therapy	[161]
EP‐NKCOF‐73	post‐synthesis modification	Lipase	surface attachment	biocatalysis	[66]
Fe_3_O_4_@COF	composite Method	GOx	surface attachment	pollutant removal	[172]
pSC4‐AuNPs@COFs	composite Method	HRP	surface attachment	biosensor	[180]
COFs‐PB	bottom‐up Strategy	HRP	pore confinement	biosensor	[70]
Fe‐COF‐H3	post‐synthesis modification	UOx	surface attachment	biosensor	[63]
*cm*COF‐TATP	post‐synthesis modification	Cyt *c*	pore confinement	biocatalysis	[187]
Au◎COFcap‐2	composite Method	ω‐Transaminase	in situ crystallization	biocatalysis	[71]
MNPs@BioHOF‐1	bottom‐up strategy	CAT	in situ crystallization	biosensor	[181]
Ce6@EnHOF‐101	post‐synthesis modification	GOx/CAT	in situ crystallization	therapy	[60]
TNU‐14	bottom‐up strategy	CAT/ α‐amylase	in situ crystallization	biocatalysis	[72]
PHOF	bottom‐up strategy	Lac	in situ crystallization	pollutants degradation	[188]
DS‐HOF	bottom‐up strategy	GFP	in situ crystallization	biodelivery	[189]
meso‐HOF	bottom‐up strategy	GOx/ HRP	in situ crystallization	biocatalysis	[190]

### 4.1 Enzyme carriers

Enzyme catalysis is recognized as an effective approach for obtaining enantiomerically pure compounds due to its excellent regioselectivity/chemoselectivity and catalytic efficiency.^[^ ^140^, ^141^, ^142^ ^]^ Nonetheless, direct utilization of free enzymes is a major challenge due to their low operational stability and poor recoverability.^[^ ^143^ ^]^ To address these limitations, immobilization of enzymes using functional solid carriers is a convenient strategy.^[^ ^144^, ^145^ ^]^ Such carriers not only minimize external interference with the enzymes but also stabilize their dynamic structures.^[^ ^146^ ^]^ Artificial porous materials with high porosity, large specific surface area and superior stability have received wide attention in the field of enzyme immobilization.

**Figure 7:**
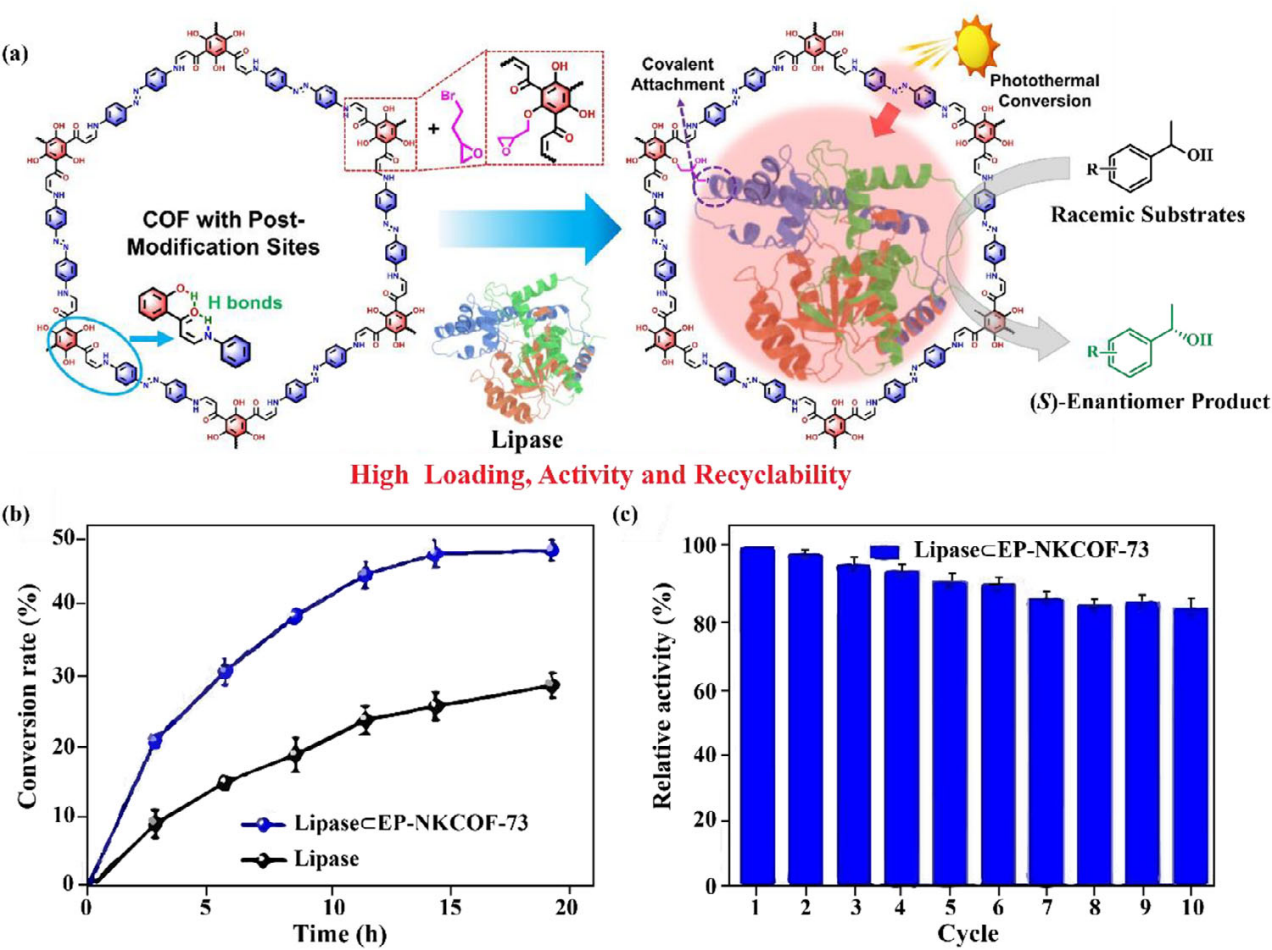
a) Schematic diagram of enzyme immobilization and catalysis. b) Conversion rate kinetics curve of free lipase and lipase⊂EP‐NKCOF‐73. c) Recyclability of lipase⊂EP‐NKCOF‐73. Reproduced with permission.^[^ ^66^ ^]^ Copyright 2024, Wiley.

Ouyang et al. successfully prepared heterogeneous biocatalysts through spatial immobilization technology, effectively addressing the critical issues of limited substrate mass transfer and enzyme conformational changes in traditional enzyme immobilization processes. The authors employed hierarchically porous NU‐1003 as the carrier, whose unique interconnected mesoporous‐microporous channel system provided an ideal environment for enzyme immobilization. By precisely modifying the mesoporous channels with fatty acid of adjustable chain lengths, efficient capture and immobilization of lipase were achieved. This design offers dual advantages: i) the interconnected multilevel pore structure ensures efficient mass transfer between the immobilized enzyme and the reaction medium; ii) the hydrophobic pore environment formed by fatty acid modification effectively induces and stabilizes the active opening conformation of lipase through interfacial interactions, resulting in higher catalytic efficiency of natural lipase.^[^ ^147^ ^]^ Chen et al. prepared substantial hydroxyl mesoporous covalent organic frameworks, which could be readily post‐modified with epibromohydrin to covalently immobilize lipase through an intramolecular hydrogen‐bonding (IMHB) activated Michael addition‐elimination reaction (**Figure** **7**a). This strategy yielded an immobilized enzyme with high loading capacity (0.52 g g⁻¹) and high activity. Additionally, the introduction of photosensitive azo groups to the COF carriers could enhance the catalytic performance of lipase through a solar‐driven photothermal promotion strategy. Under sunlight irradiation, 8 racemic substrates were catalyzed by lipase⊂EP‐NKCOF‐73 with high conversion rates approaching the theoretical value (50%) (Figure 7b). Lipase⊂EP‐NKCOF‐73 showed superior catalytic performance, emphasizing the advantages of covalent immobilization technology over free lipase and lipase@NKCOF‐73. Remarkably, the heterogeneous biocatalyst lipase⊂EP‐NKCOF‐73 maintained high catalytic activity (>80%) after 10 catalytic cycles (Figure 7c), demonstrating the excellent reusability.^[^ ^66^ ^]^ In another work, the covalent immobilization of Cyt *c* onto EP‐TD‐COFs was investigated by Wang et al. Resultingly, covalent interactions altered the secondary structure of Cyt *c* and increased the accessibility of the heme center, thereby improving the catalytic activity. Furthermore, the stability of covalently anchored Cyt *c* was significantly improved due to confinement effects and interfacial interactions.^[^ ^118^ ^]^

### 4.2 Photocatalysis

#### 4.2.1 Reduction of CO_2_

**Figure 8:**
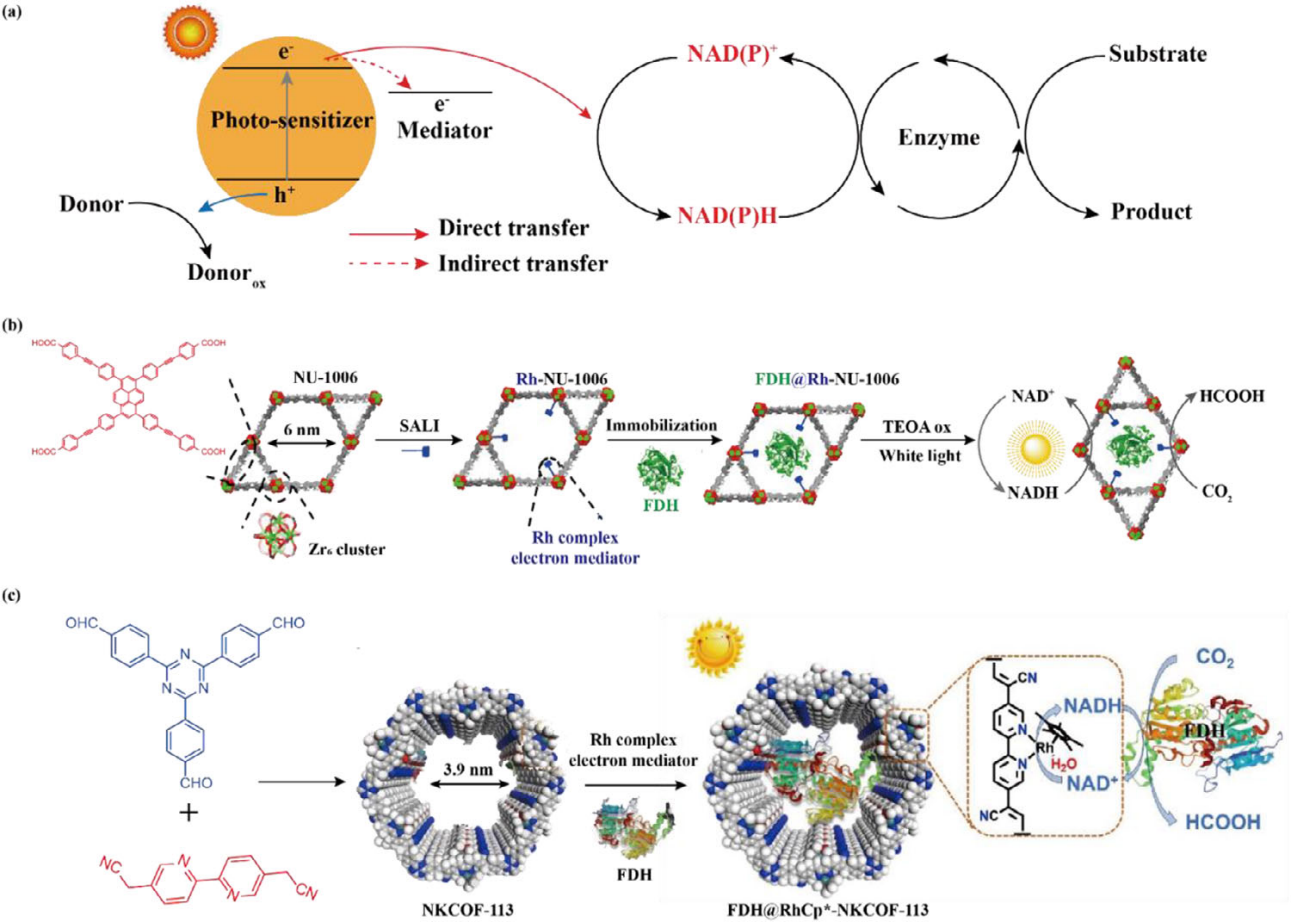
a) Schematic diagram of photoenzymatic cascade catalysis for reduction of CO_2_. b) MOF‐based multifunctional biological nanoreactors for CO_2_ reduction. Reproduced with permission.^[^ ^51^ ^]^ Copyright 2020, American Chemical Society. c) COF‐based multifunctional biological nanoreactors for CO_2_ reduction. Reproduced with permission.^[^ ^58^ ^]^ Copyright 2022, Wiley.

With the rapid development of human society, continuously increasing carbon dioxide (CO_2_) emissions pose a serious challenge to energy and the environment.^[^ ^148^ ^]^ CO_2_ conversion using renewable energy sources, such as solar energy, offers new opportunities to reduce CO_2_ levels in the atmosphere, while also producing high value‐added compounds (e.g., formic acid, methane, formaldehyde and methanol).^[^ ^149^ ^]^ Early photocatalysts for CO_2_ conversion, including metal oxides and conjugated microporous polymers,^[^ ^150^, ^151^ ^]^ produced mixed products containing CO, CH_4_, CH_3_OH, and HCOOH. To selectively convert CO_2_ to specific products, researchers employed enzymes to reduce CO_2_, which requires the acquisition of external electrons through redox reactions for its enzymatic conversion.^[^ ^152^ ^]^ Typically, artificial porous materials not only immobilize enzymes, but also act as photocatalysts to provide these electrons. The process involves three essential steps: First, the photocatalyst absorbs photons with energy greater than its bandgap, exciting electrons from the valence band to the conduction band, resulting in the creation of photogenerated electrons and holes. Second, the photogenerated electrons migrate to the surface of photocatalyst and bind to the coenzyme (NAD^+^/NADP^+^). Finally, the coenzyme (NAD^+^/NADP^+^) gains two electrons and binds to a proton to produce reduced coenzyme (NADH/NADPH), which selectively reduces CO_2_ in the presence of the enzyme (**Figure** **8**a).^[^ ^153^ ^]^

Studies have shown that formate dehydrogenase (FDH) and Cp*Rh can be effectively co‐immobilized on MOFs and COFs. Not only do these multifunctional bio‐nanoreactors significantly enhance the stability of formate dehydrogenase, but they also improve the regeneration of coenzymes. For instance, Farha and co‐authors designed a mesoporous MOF material NU‐1006 with photocatalytic properties. Subsequently, NU‐1006 was utilized to immobilize FDH and the electron mediator Cp*Rh (2,2′‐bipyridyl‐5,5′‐carboxylic acid)Cl. Under light irradiation, the system efficiently converted CO_2_ to formic acid (Figure 8b). The electron‐mediator Cp*Rh (2,2′‐bipyridyl‐5,5′‐dicarboxylic acid)Cl was immobilized on NU‐1006 to promote ultrafast electron transfer upon irradiation, in which Rh‐NU‐1006 regenerated NAD^+^ at a rate of ≈28 mM h^−1^. Especially, FDH@Rh‐NU‐1006 also converted CO_2_ to formic acid using reduced coenzymes with a selectivity of up to 865 h^−1^ over 24 h.^[^ ^51^ ^]^ In another study, Han and co‐authors designed a photocatalytic MOF (NU‐1006) and co‐immobilized the electronic mediator 1‐(carboxynonyl)‐1′‐methyl‐[4,4′‐bipyridine]‐1,1′‐diium (CNMV) with FDH for the reduction of CO_2_. With this versatile photo‐enzymatic nanoreactor, the CNMV‐NU‐1006 was able to deliver photogenerated electrons to the enzyme under light irradiation in a rapid and sustained manner. As a result of this feature, a photocatalytic CO_2_ reduction rate of 1.17 mmol h^−1^ per unit of enzyme was achieved, a rate that represents a 190‐fold increase in reaction kinetics compared to the conventional use of NADH as a coenzyme.^[^ ^61^ ^]^

Mesoporous COFs have also been reported as supports for immobilized FDH and electron mediator. Chen et al. constructed a novel mesoporous olefin‐linked COF material, which was used for the first time for immobilizing FDH and Rh electronic mediators (Figure 8c). In this case, the Rh‐based electron mediator was anchored to NKCOF‐113 via coordination bonds. By tailoring the content of Rh electron mediator, the regeneration of coenzymes was enhanced with an apparent quantum yield of 9.17 ± 0.44%, which exceeded all previous NADH regeneration photocatalysts based on porous organic materials. Finally, FDH was immobilized on RhCp*‐NKCOF‐113 by electrostatic interactions.^[^ ^58^ ^]^ Exceptionally, such versatile photo‐enzymatic nanoreactors selectively convert CO_2_ to formic acid with high efficiency and reusability.

#### 4.2.2 Chiral Synthesis

The development of artificial porous materials has led to new trends in other photocatalytic applications, such as the Mannich reactions, dynamic kinetic resolution of amines, and reduction of ketones. For photocatalysts, critical reaction intermediates are generated under light irradiation,^[^ ^154^ ^]^ and subsequently cascaded with enzymes to complete the reaction. Thus, immobilization of enzymes in porous materials facilitates the establishment of new green and sustainable chemical processes. The principal steps in the photocatalytic reaction that produce the crucial intermediate products are: 1) Substrates are adsorbed by the material; 2) Under light irradiation, the photocatalytic metals in the material are excited to yield electrons and holes; 3) Charges are separated and transferred to the surface of the photocatalyst to participate in the redox reaction and generate the crucial intermediate products of the reaction.^[^ ^155^ ^]^ Therefore, the cascade reaction of biocatalysis and chemocatalysis can be accomplished by a well‐designed multifunctional bio‐nanoreactor, which significantly improves the catalytic capacity.

**Figure 9:**
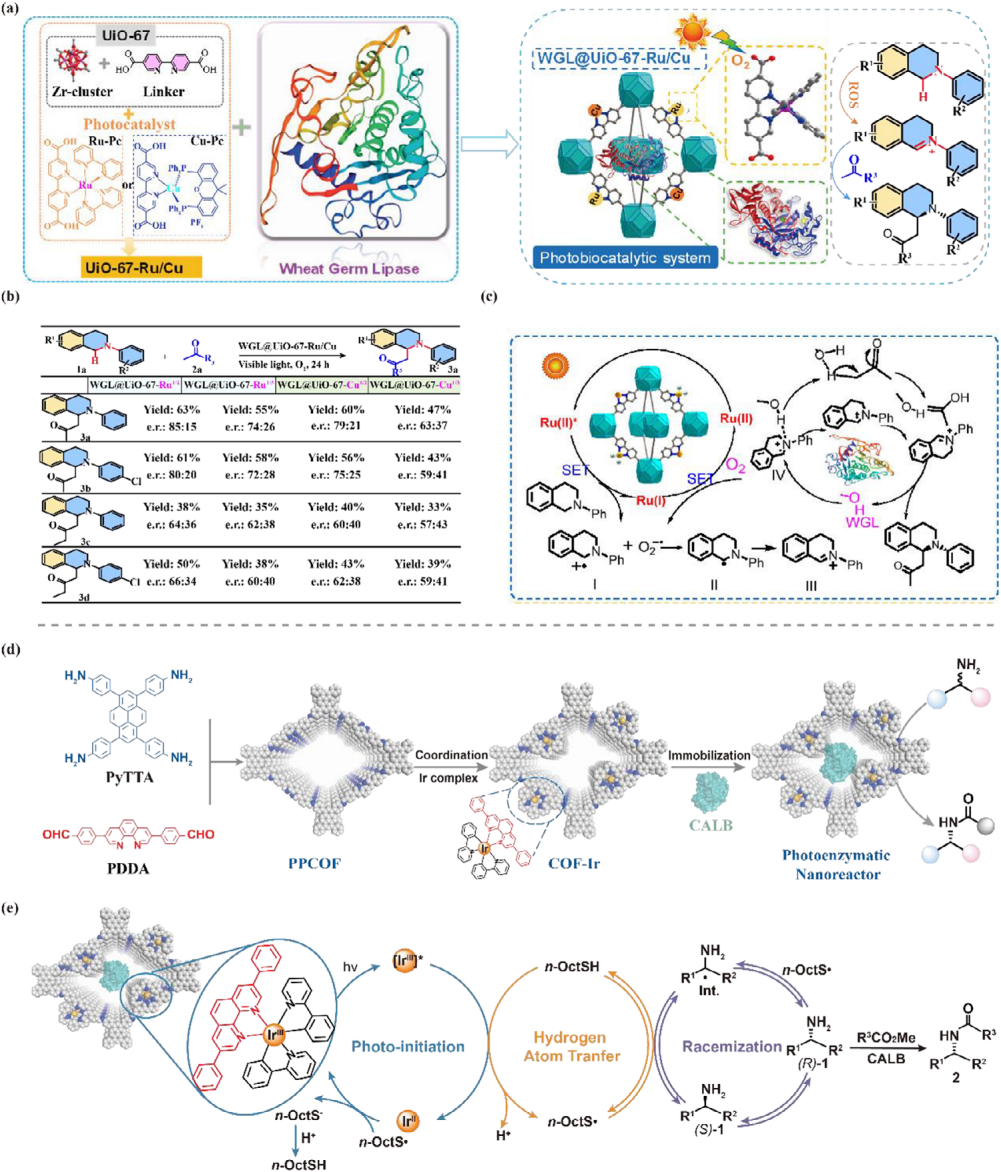
a) Schematic diagram of MOF‐based photoenzymatic enantioselective catalysis. b) WGL@UiO‐67‐Ru/Cu synthesis of N‐aryl‐substituted tetrahydroisoquinoline. c) Photoenzymatic catalysis mechanism diagram of WGL@UiO‐67‐Ru/Cu. Reproduced with permission.^[^ ^65^ ^]^ Copyright 2024, American Chemical Society. d) Multifunctional photoenzymatic nanoreactor for dynamic kinetic resolution of amines. e) Possible catalytic mechanism of CALB@COF‐Ir. Reproduced with permission.^[^ ^67^ ^]^ Copyright 2024, Wiley.

There have been several reports for photoenzymatic cascade catalysis synthesis of chiral compounds by multifunctional biological nanoreactors. Combining photocatalysis with biocatalysis, Chen's research group synthesized a Zr‐based MOF material of UiO‐67, in which the photocatalyst and wheat germ lipase (WGL) were encapsulated for asymmetric catalytic reactions via a mechanochemical synthesis strategy (**Figure** **9**a). Subsequently, the versatile photoenzymatic nanoreactor was applied to the asymmetric Mannich reactions, which exhibited excellent photobiological activity, operational stability and reusability. In addition, the photo‐biocatalyst with a wide range of substrates successfully synthesized asymmetric *N*‐aryl‐substituted tetrahydroisoquinolines (Figure 9b). Based on the experimental results, a probable internal mechanism for the synthesis of *N*‐aryl‐substituted tetrahydroisoquinolines by a multifunctional bio‐nanoreactor was proposed by the authors, as shown in Figure 9c. The reaction consists of two pathways: single electron transfer (SET) and energy transfer (ET). In the presence of light, Ru(II) was converted to Ru(II)*, followed by the reduction of the substrate by Ru(II)* to produce intermediates I and Ru(I). Intermediate I was then coupled with superoxide anion O_2_^•−^ to form intermediate II, which was then converted by proton transfer to III and further converted to IV. Afterwards, intermediate IV underwent an asymmetric catalytic reaction with acetone catalyzed by WGL to produce the product.^[^ ^65^ ^]^ Meanwhile, Chen's group has designed and constructed a multifunctional photo‐enzymatic nanoreactor WGL@NKCOF‐118(M) (M = H, Zn, Cu, Ni) for asymmetric Mannich reactions. In this photoenzymatic integrated system, the proximity effect between the photocatalyst and enzyme ensures excellent selectivity for radical conversion during substrate binding. Under oxygen and light conditions, the metal‐containing photocatalytic materials can smoothly generate reaction intermediates and complete the reaction under the action of enzymes. The prepared WGL@NKCOF‐118 was characterized by excellent reactivity and reusability, with the activity remaining above 95% after several cycles. More importantly, the asymmetric reaction to form C(sp^3^)‐C(sp^3^) bonds could not be realized by WGL or COFs alone.^[^ ^59^ ^]^

Teng's group has reported a novel multifunctional photoenzymatic nanoreactor of CALB@COF‐Ir, which co‐immobilized Ir(ppy)_2_ complex and Candida antarctica lipase B (CALB) on a light‐sensitive covalent organic framework, with application to asymmetric catalysis (Figure 9d). Specifically, the dynamic kinetic resolution (DKR) of secondary amines led to the preparation of a series of chiral amine compounds with yields up to 99% and enantioselectivities up to 99%. In this system, COF‐Ir not only acted as a protective shell to prevent the inactivation of CALB, but also promoted the racemization of secondary amines through the photo‐induced hydrogen atom transfer (HAT) process (Figure 9e). Photoelectric characterization and TDDFT calculations indicated that the introduction of Ir(ppy)_2_ units played a crucial role in the photocatalytic performance of COFs, which was enhanced by promoting the separation of photogenerated electron‐hole pairs. In addition, the multifunctional photo‐enzyme nanoreactor maintained good catalytic performance after five cycles of use, which was highly enantioselective, stable and reproducible.^[^ ^67^ ^]^ For bimetallic modified artificial porous materials, each metal plays a different role in the material and catalyzes synergistically with enzymes. For instance, Huang and co‐workers co‐immobilized Ru(N^N)_3_, Cp*Rh and *Re*ADH to prepare *Re*ADH@Ru/Rh‐COF, which was used for the production of 1‐(propylthio)pentan‐3‐ol. Under the catalytic reaction of Ru‐COF, 1‐propanethiol and 1‐penten‐3‐one underwent a thiol‐ene click reaction to produce prochiral ketones. Furthermore, Rh‐COF photo‐regenerated NADH from NAD^+^. Finally, 1‐(propylthio)pentan‐3‐ol was obtain in the presence of the enzyme, giving a product with high yield and high enantioselectivity. Without the enzyme or Ru/Rh, this reaction could not be completed.^[^ ^156^ ^]^ This study further demonstrates the advantages of immobilizing enzymes in artificial porous materials.

#### 4.2.3 Photodynamic Therapy

Cancer is one of the diseases that pose a serious threat to human health, so finding more effective treatments has been a top priority for researchers. Photodynamic therapy that utilizes light, oxygen and photosensitizers to generate reactive oxygen species (ROS) to induce cell death has been widely adopted as a cancer treatment.^[^ ^157^ ^]^ In general, the photodynamic therapy consists of three main components: First, the photosensitizer is excited by laser. Second, oxygen within the tissues is activated by the photosensitizer. Finally, the oxygen produces free radicals and singlet oxygen, which kills the cancer cells. Nevertheless, the effectiveness of such approach relies on the availability of suitable carriers for drug delivery.^[^ ^158^, ^159^ ^]^ MOFs, COFs and HOFs, which are known for their excellent biocompatibility, have been employed in this field.

**Figure 10:**
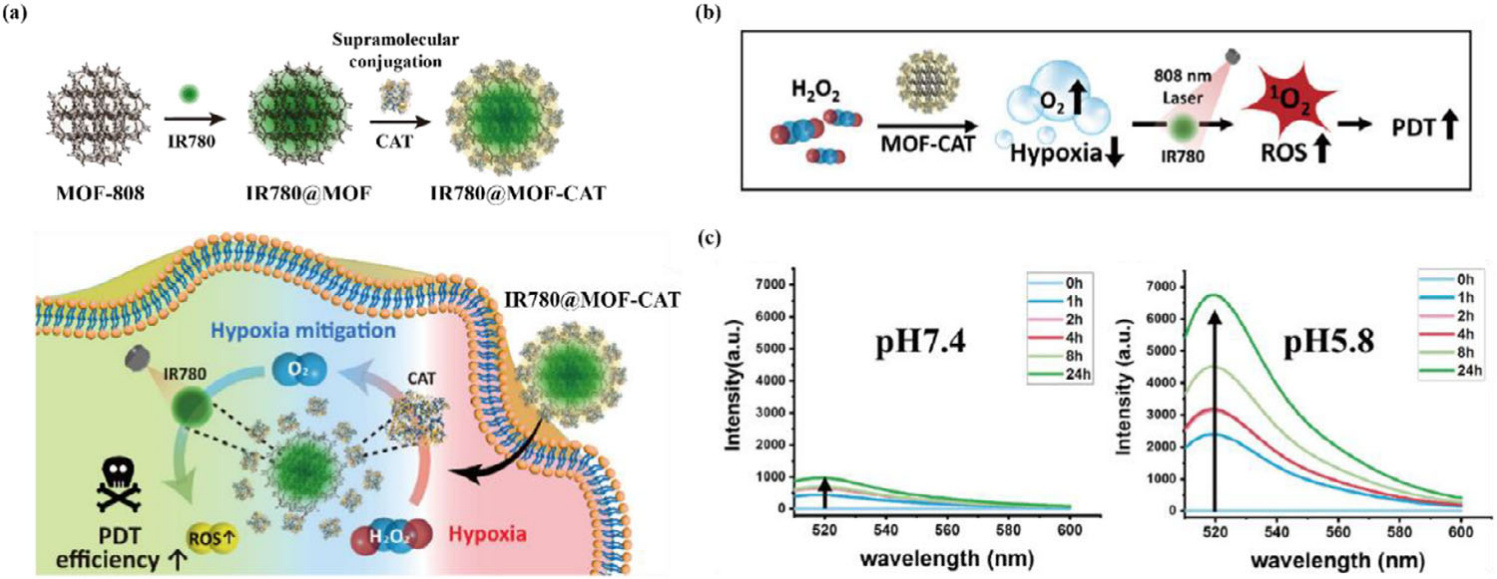
a) Schematic diagram of MOF‐based multifunctional biological nanoreactors for photodynamic therapy. Reproduced with permission. b) Schematic diagram of photodynamic therapy enhancement. c) FL emission of the MOF‐detached CAT in neutral or acidic PBS. Reproduced with permission.^[^ ^163^ ^]^ Copyright 2023, American Chemical Society.

Recently, artificial porous materials with customized compositions have emerged as ideal candidates for cancer treatment.^[^ ^160^, ^161^, ^162^ ^]^ Beyond acting as photosensitizers to generate cytotoxic reactive oxygen species (ROS), such materials address a key challenge in photodynamic therapy: porous structures can enhance the supply of oxygen to hypoxic tumor tissues, thereby synergistically improving therapeutic efficacy. Ryu and co‐authors constructed a multifunctional photo‐enzymatic nanoreactor called IR780@MOF‐CAT, which served as a photosensitizer as well as an oxygen deliverer. Multifunctional photo‐enzymatic nanoreactor was prepared by co‐immobilizing the photosensitizer IR780 and enzyme catalase (CAT) with MOF‐808 (**Figure** **10**a). In this effort, CAT‐loaded MOF NPs were able to successfully penetrate into hypoxic cancer cells and synergize with IR780 to effectively trigger oxygen generation and photodynamic therapy processes. In terms of therapeutic efficacy, IR780@MOF‐CAT was validated in vitro using HeLa cancer cells. In acidic cancer cells, the protonated MOF‐CAT was dissociated from each other, with free catalase successfully alleviating cellular hypoxia and promoting ROS production by the photosensitizer IR780, which in turn enhanced photodynamic therapy (Figure 10b). Furthermore, as shown in Figure 10c, FL emission was measured with acidic PBS and was found to increase in fluorescence intensity with time, whereas no significant FL emission was detected in alkaline PBS.^[^ ^163^ ^]^

COFs and HOFs also hold great promise for cancer therapy. For example, Tang's group engineered a novel enzyme nanopocket using a porphyrin‐based COF as the structural unit, then co‐immobilized GOx and CAT for use in photodynamic therapy and long‐term starvation therapy. As a result, glucose in cancer cells was catalyzed to break down, thereby cutting off the nutrient supply. Subsequently, H_2_O_2_ derived from the catalytic decomposition of glucose was converted to O_2_ by CAT catalytic action, which further improved the efficiency of glucose depletion and facilitated the starvation therapy. More interestingly, the immobilization of enzyme by COF prolonged the residence time in tumor tissue, thus facilitating long‐term starvation therapy. Simultaneously, ROS produced by porphyrins in COF effectively eliminated cancer cells under light irradiation, reaching the purpose of photodynamic therapy.^[^ ^161^ ^]^ A HOF‐based biocomposite with photocatalytic properties was developed by Wang et al. Catalase@RuB‐HOFs were synthesized using catalytically active tris(4,4′‐dicarboxylicacid‐2,2′‐bipyridyl)ruthenium(II) self‐assembled with [1,1′‐biphenyl]‐4,4′‐biscarboximidamide and encapsulated with catalase. This biocomposite served as both a bioorthogonal chemical catalysts for the production of H_2_S and an enzymatic catalyst for the decomposition of H_2_O_2_. Besides, it exhibited significant neuroprotective effects against oxidative stress. Collectively, catalase@RuB‐HOFs afford a new approach for the treatment of diseases associated with cellular oxidative stress.^[^ ^64^ ^]^

### 4.3 Chemical Catalysts

#### 4.3.1 Dynamic Kinetic Resolution

Dynamic Kinetic Resolution (DKR) is an effective method for the preparation of chiral pharmaceutical intermediates by converting racemic samples into single enantiomers with 100% atom economy.^[^ ^164^ ^]^ Immobilization of enzymes for organocatalysis using well‐designed artificial porous materials offers several advantages: i) Multifunctional bio‐nanoreactors exhibit well dispersion, which improves substrate accessibility; ii) Metal NPs or enzymes immobilized on the surface of porous materials are unable to clog the pores and provide accessible active sites. Therefore, investigators designed artificial porous materials with different structures for dynamic kinetic resolution.

**Figure 11:**
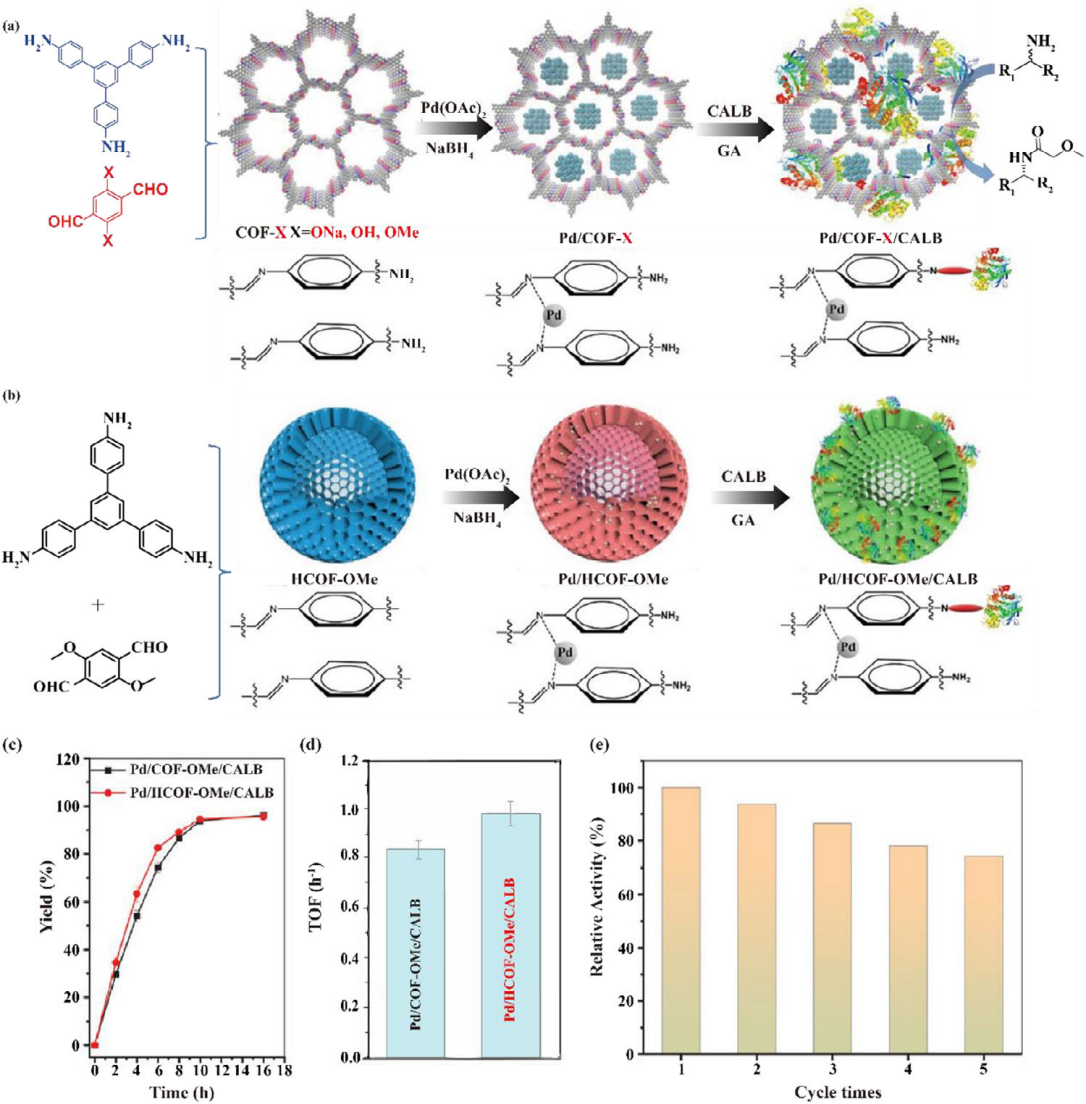
a) Synthesis diagram of Pd/COF‐X/CALB and dynamic kinetic resolution of amines. b) Synthesis diagram of hollow structure Pd/HCOF‐OMe/CALB. c) Reaction process and d) corresponding turnover frequency (TOF) of DKR of 1‐phenethylamine catalyzed by Pd/COF‐OMe/CALB and Pd/HCOF‐OMe/CALB. e) Recyclability of Pd/HCOF‐OMe/CALB. Reproduced with permission.^[^ ^79^ ^]^ Copyright 2024, Wiley.

In 2024, Jiang and co‐works designed a series of COFs with different functional groups (COF‐ONa, COF‐OH, and COF‐OMe). Then, palladium nanoparticles (Pd NPs) and CALB were co‐immobilized in the pores and surfaces of these COFs to obtain Pd/COF‐X/CALB (X = ONa, OH, OMe) catalysts. Afterwards, the prepared multifunctional biological nanoreactors were employed for the DKR of primary amines (**Figure** **11**a), with COF‐OMe showing the highest catalytic activity, followed by COF‐OH and COF‐ONa. Significantly, the hydrophobicity of COF‐OMe altered the secondary structure of CALB, exposing the active sites and improving its catalytic performance. In addition, the hydrophobicity of COF‐OMe promoted the enrichment of the catalyst for the substrate, which further improved the catalytic activity. Simultaneously, Pd/HCOF‐OMe/CALB was obtained by using COF with a hollow structure as the carrier material (Figure 11b), which increased the accessible active sites and mass transfer efficiency (Figure 11c,d). High yields (90%–95%) and ee values (87%–97%) were obtained for primary amines prepared with Pd/HCOF‐OMe/CALB. After 5 cycles, Pd/HCOF‐OMe/CALB still maintained 68% activity (Figure 11e).^[^ ^79^ ^]^

#### 4.3.2 Degradation of Pollutants

**Figure 12:**
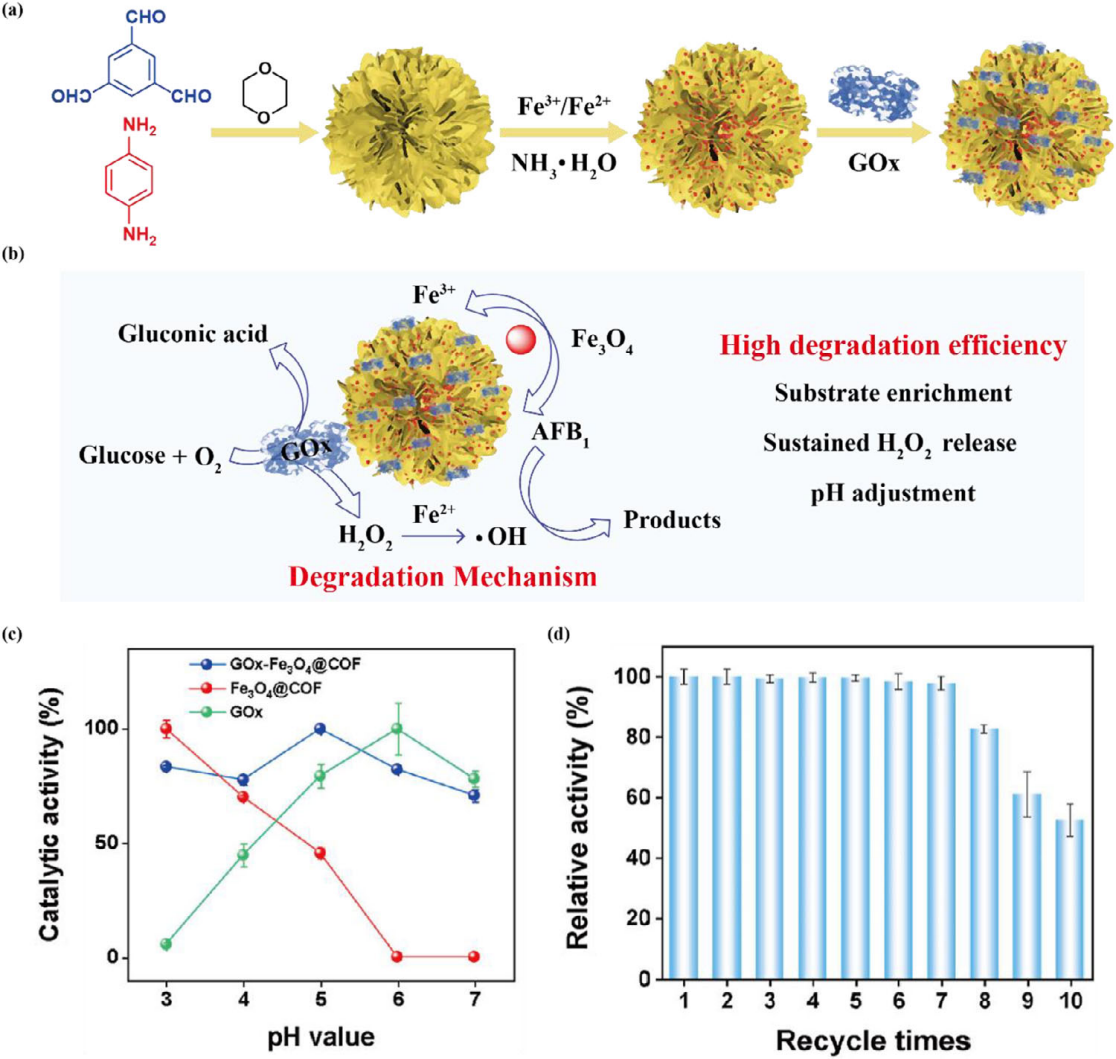
a) Schematic diagram for the synthesis of GOx‐Fe_3_O_4_@COF. b) Degradation of AFB_1_ by GOx‐Fe_3_O_4_@COF. c) Effects of pH on the catalytic activity of GOx‐Fe_3_O_4_@COF, Fe_3_O_4_@COF and GOx. d) Recyclability of GOx‐Fe_3_O_4_@COF. Reproduced with permission.^[^ ^172^ ^]^ Copyright 2023, American Chemical Society.

In recent years, food safety has garnered widespread attention due to a variety of hazard factors, encompassing common contaminants such as antibiotics, foodborne pathogens, mycotoxins, heavy metals, pesticide residues, and aromatic hydrocarbons.^[^ ^165^, ^166^, ^167^, ^168^ ^]^ Conventional methods for contaminant degradation are susceptible to secondary contamination, residual reagents or materials, and specific equipment required.^[^ ^169^, ^170^, ^171^ ^]^ Researchers have identified a chemo‐enzymatic cascade method for efficient and green removal of environmental pollutants. Multifunctional bio‐nanoreactors prepared by cascading artificial porous materials with specific functions and enzymes can efficiently remove pollutants and realize recycling. For example, Li and co‐authors successfully prepared a novel enzyme‐metal hybrid catalyst by co‐immobilizing GOx and Fe_3_O_4_ NPs on a flower‐shaped COF (**Figure** **12**a). The toxin was not only adsorbed by the multifunctional bioreactor, but also degraded with the synergistic catalytic effect of GOx and Fe_3_O_4_ NPs. Subsequently, the prepared GOx‐Fe_3_O_4_@COF was employed for the adsorption and degradation of aflatoxin B_1_ (AFB_1_) (Figure 12b). In the GOx‐Fe_3_O_4_@COF‐catalyzed chemoenzymatic cascade reaction, the degradation efficiency of AFB1 was 3.5 times higher than that of Fe_3_O_4_@COF ((Figure 12c). More importantly, the GOx‐Fe_3_O_4_@COF hybrid catalyst displayed high activity over a wide pH range of 3.0–7.0, eliminating the pH limitation of the Fenton reaction. After 10 cycles, the GOx‐Fe_3_O_4_@COF still preserved 52% of the residual activity (Figure 12d), indicating that the multifunctional bio‐nanoreactor features high stability, catalytic activity and recyclability.^[^ ^172^ ^]^

### 4.4 Sensing

Biosensors are emerging as one of the common analytical methods for the detection of pollutants due to their high sensitivity, selectivity and adaptability to in situ measurements.^[^ ^173^, ^174^ ^]^ Function‐based artificial porous materials can be applied to biosensing for the following reasons: 1) Artificial porous materials feature excellent structural superiority to improve the efficiency of enzyme encapsulation. 2) Signal amplification is facilitated by the introduction of metal nanoparticles into artificial porous materials. 3) Unique structure of the artificial porous material contributes to the prevention of material clumping. 4) Multifunctional bio‐nanoreactors prepared by immobilizing enzymes using artificial porous materials improve sensing sensitivity through synergistic catalysis among their components.^[^ ^175^, ^176^ ^]^ As a result, over the past few years, there has been a strong interest in multifunctional bio‐nanoreactors for biosensing applications.

**Figure 13:**
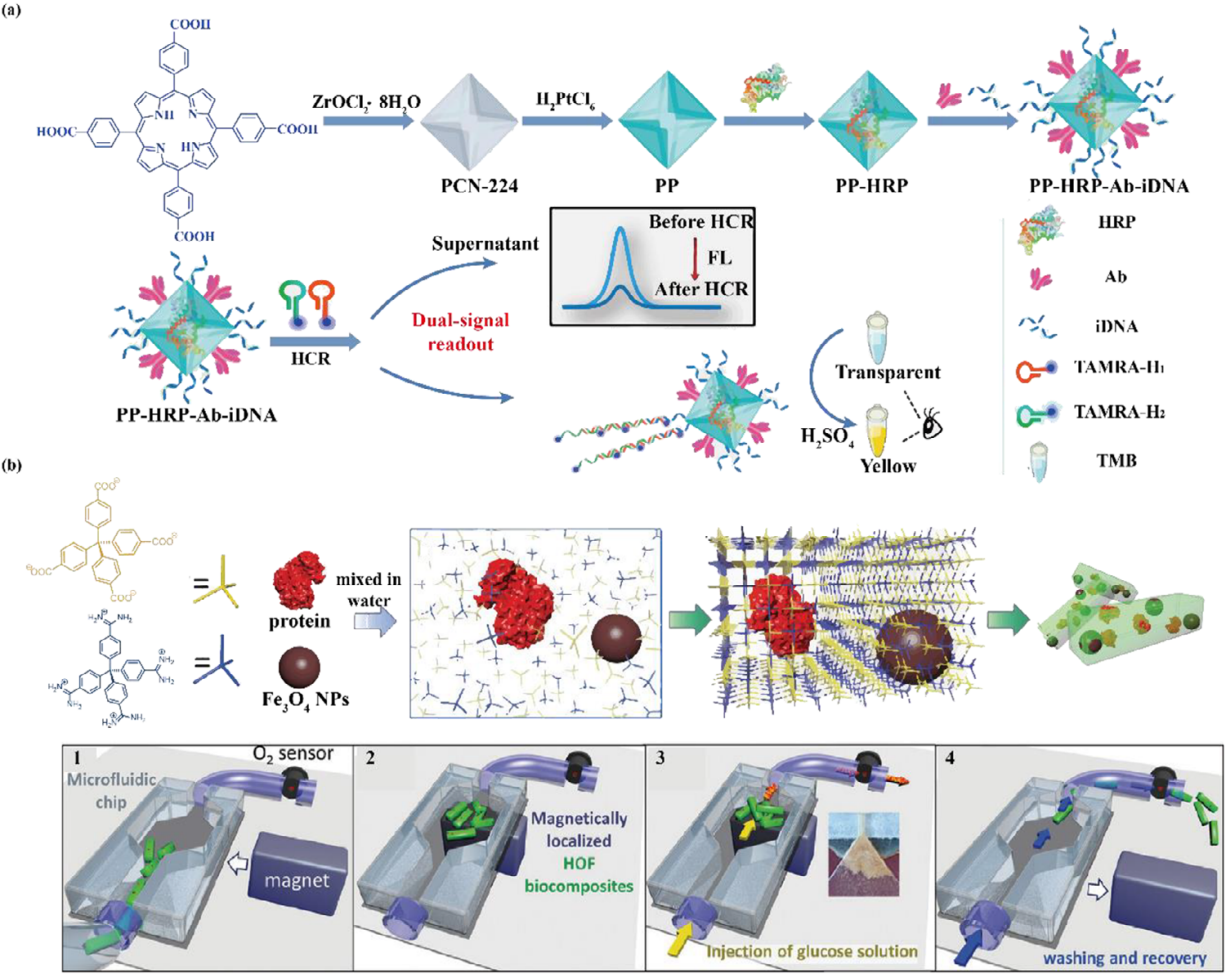
Schematic diagram of preparing the biosensor based on multifunctional biological nanoreactor for detection of a) aflatoxin B_1_ and *Salmonella enteritidis*. Reproduced with permission.^[^ ^178^ ^]^ Copyright 2022, American Chemical Society. b) deoxynivalenol. Reproduced with permission.^[^ ^181^ ^]^ Copyright 2023, ScienceDirect.

Horseradish peroxidase (HRP) and glucose oxidase (GOx) have been extensively exploited as biocatalysts in biosensors to catalyze the chromogenic reaction of tetramethylbenzidine (TMB). However, the poor stability of free enzyme leads to low reproducibility and sensitivity of biosensors.^[^ ^177^ ^]^ Well‐conceived multifunctional bio‐nanoreactors can improve sensitivity while enhancing enzyme stability. Chen's group prepared MOFs (PCN‐224) for the co‐immobilization of Pt NPs and HRP, which were applied to the detection of food toxins (**Figure** **13**a). Specially, the synergistic catalytic effect between Pt NPs and HRP was revealed, dramatically improving the performance of the biosensor. In practical applications, the biosensor showed high sensitivity, with detection limits of 0.65 pg mL^−1^ for aflatoxin B_1_ and 4 CFU mL^−1^ for *Salmonella enterica*, demonstrating its potential for food safety monitoring.^[^ ^178^ ^]^ Meanwhile, Chen's group also successfully realized the detection of deoxynivalenol with high sensitivity and low detection limit by co‐immobilizing Pt NPs and GOx with PCN‐224.^[^ ^179^ ^]^

Due to their excellent structural properties, COFs and HOFs hold great potential for applications in biosensing. Li's group designed and prepared a multifunctional bio‐nanoreactor named HRP‐pSC4‐AuNPs@COFs, in which spherical COFs was used to co‐immobilize *para*‐sulfocalix arene hydrate (pSC4)‐modified AuNPs and HRP. Subsequently, the multifunctional bio‐nanoreactor was applied to the electrochemical detection of colorectal cancer (CRC)‐derived exosomes. In this system, pSC4 selectively recognized and bound to various amino acid residues on the exosome surface, while AuNPs accelerated the transfer of charge carriers and improved the response speed of biosensors. High porosity of the COF enabled the loading of a large amount of HRP, which conferred high catalytic activity to the COF and significantly improved the stability of HRP. For the detection of CRC‐derived exosomes, the biosensor demonstrated excellent analytical performance with a detection limit of 160 particles µL^−1^.^[^ ^180^ ^]^ Falcaro's group employed a one‐pot synthesis method to prepare a multicomponent biocomposite, MNPs‐enzyme@BioHOF‐1. Enzymes (CAT and GOx) and magnetic nanoparticles (MNPs) were co‐immobilized on HOF‐1. Incorporation of MNPs into the biocomposite strengthened the enzymatic activities of CAT and GOx, along with endowing them with dynamic localization capabilities (Figure 13b). In addition, MNPs‐GOx@BioHOF‐1 was utilized to fabricate glucose microfluidic biosensors with high stability (37% retention after 1200 cycles) and linear response.^[^ ^181^ ^]^

## 5 Summary and Outlook

In the realm of biocatalysis, the emergence of artificial porous materials marks a major paradigm shift, providing innovative solutions to the enduring challenges of enzyme immobilization. Through a comprehensive review, this paper elucidates the profound impact of these porous materials on enzyme stability, functionality, and recyclability. Compared with free enzymes, multifunctional bio‐nanoreactors constructed from artificial porous materials not only create favorable conditions for diversified application of enzymes, but also highlight their functional applications as enzyme carriers, photocatalysts, and chemical catalysts. Formulating artificial porous materials through bottom‐up strategies, post‐synthetic modification and composite approaches is essential for the development of advanced biocomposites for a wide range of applications.

Conventionally, the cascade reaction conditions between enzyme and catalyst are complicated to manage, making the enzyme easy to be inactivated and not recyclable, which results in high cost. In view of the fact that artificial porous materials can play the dual roles of enzyme carrier and catalyst at the same time, this paper reviewed the excellent performance of artificial porous materials as multifunctional carriers for immobilizing enzymes from the aspects of enhancing enzyme activity and enriching enzyme reactivity. In addition, the application of artificial porous materials as photocatalysts for enzyme immobilization in therapeutics, particularly in innovative approaches to cancer treatment such as photodynamic therapy and starvation therapy, emphasizes their potential to improve patient prognosis. The chemoenzymatic cascade facilitated by frameworks provide effective solutions for contaminant detection with sensitivities that exceed conventional methods. In order to better exploit multifunctional bio‐nanoreactors, the following pressing issues must be considered:

1. Novel immobilization method: Enzyme immobilization is a crucial factor in preserving enzyme stability in porous materials. In addition to surface immobilization and pore encapsulation, combining these methods is a promising immobilization strategy, such as immobilizing enzymes by covalent bonding and pore infiltration. Not only can this method further improve the stability of the enzyme, but it also prevents the enzyme from leaking during the reaction. Simultaneously, this facilitates the enzyme to perform various biochemical reactions in complex matrices.
2. Enhanced material design: Future investigations should emphasize the developing of MOFs, COFs, and HOFs with customized pore structures and surface functionalities, accommodating a wider range of enzymatic and biochemical reactions. Including the concept of designing materials with optimal pore sizes, shapes, and surface chemistries that can selectively interact with specific enzymes, thereby enabling improved efficiency and selectivity of enzymatic reactions. Furthermore, investigations should be extended to realize more types of asymmetric catalytic reactions (not limited to dynamic kinetic and Mannich reactions) with the aim of enhancing the potential applications of the materials in the field of biocatalysis.
3. Integration of advanced functionality: The integration of additional functional groups, nanoparticles, and hybridization of MOFs, COFs, and HOFs to create artificial porous materials is promising. With the introduction of photocatalytic sites, magnetic nanoparticles or other components, new reaction pathways can be realized, which in turn improve reaction rates or facilitate targeted drug delivery and biosensor applications. For instance, combining the catalytic properties of an artificial porous material with an enzyme could lead to the creation of an optical immunosensor for the detection of antibiotics. Noticeably, surface modification of artificial porous materials with metals allows for an increase in the concentration of oxygen vacancies, which results in the generation of reactive oxygen species that catalyze the colorimetric reaction of TMB. Furthermore, there is effective enrichment of horseradish peroxidase (HRP) by the porous material for synergistic catalysis and dual signal amplification, which better satisfies the requirements of trace target detection.^[^ ^166^ ^]^ By precisely manipulating the distribution of functional groups and the size of nanoparticles, it is also possible to achieve subtle control over reaction selectivity, which is particularly important for the synthesis of compounds with specific structures and functions.
4. Multiple catalytic activities: Artificial porous materials are capable of serving as efficient photocatalysts, chemical catalysts and electrocatalysts, while also being designable into advanced material systems with multiple catalytic activities through functional integration strategies. Future studies shall focus on the exploitation of multifunctional artificial porous materials, especially composites with both photocatalytic and chemocatalytic properties, and their application in the field of enzyme immobilization. Exceptionally, this approach is expected to achieve synergistic effects in terms of highly sensitive pollutant detection and efficient photocatalytic degradation. What's more, artificial porous materials with multiple catalytic activities can construct efficient cascade catalytic systems with enzymes,^[^ ^156^ ^]^ which not only effectively solves the technical bottleneck of traditional enzyme catalytic singularity, but also supplies innovative solutions for expanding the application scope of enzymatic reactions, demonstrating a promising application prospect in many fields.
5. Expanding applications in biomedicine and environmental science: There is a demand to explore the potential of artificial porous materials in emerging areas, such as precision medicine, where they can be used in personalized drug delivery systems. In environmental science, artificial porous materials might serve a role in sensing pollutants or catalyzing green chemistry processes. Intentionally, MOFs, COFs and HOFs can be designed as highly efficient drug carriers to achieve precise drug delivery to specific disease targets, thereby improving therapeutic efficacy and reducing side effects. Multifunctional bio‐nanoreactors can be employed as highly efficient sensors to rapidly and accurately identify and quantify hazardous substances in the environment, with great potential for the detection and treatment of pollutants. Consequently, the development of biocompatible and biodegradable materials for such applications is particularly important to ensure safety and efficacy.

In summary, the combination of artificial porous materials and enzyme immobilization techniques provides fertile ground for future research and development. Challenges such as optimizing material design and improving the efficiency of the immobilization process are not insurmountable. Future investigations on MOFs, COFs and HOFs should be concentrated on tailored material design for enzyme specificity, integration of advanced functionality to extend reactivity, and exploration of new applications in biomedical and environmental sciences, with special emphasis on biocompatibility and sustainability.

## Conflict of Interest

The authors declare no conflict of interest.
